# Ca_v_3 T-Type Voltage-Gated Ca^2+^ Channels and the Amyloidogenic Environment: Pathophysiology and Implications on Pharmacotherapy and Pharmacovigilance

**DOI:** 10.3390/ijms23073457

**Published:** 2022-03-22

**Authors:** Anna Papazoglou, Muhammad Imran Arshaad, Christina Henseler, Johanna Daubner, Karl Broich, Jürgen Hescheler, Dan Ehninger, Britta Haenisch, Marco Weiergräber

**Affiliations:** 1 Experimental Neuropsychopharmacology, Federal Institute for Drugs and Medical Devices (Bundesinstitut für Arzneimittel und Medizinprodukte, BfArM), Kurt-Georg-Kiesinger-Allee 3, 53175 Bonn, Germany; anna.papazoglou@bfarm-research.de (A.P.); imran_ucp@yahoo.com (M.I.A.); christina.henseler@bfarm-research.de (C.H.); johanna.daubner@bfarm-research.de (J.D.); 2 Federal Institute for Drugs and Medical Devices (Bundesinstitut für Arzneimittel und Medizinprodukte, BfArM), Kurt-Georg-Kiesinger-Allee 3, 53175 Bonn, Germany; karl.broich@bfarm.de (K.B.); britta.haenisch@bfarm.de (B.H.); 3 Faculty of Medicine, Institute of Neurophysiology, University of Cologne, Robert-Koch-Str. 39, 50931 Cologne, Germany; j.hescheler@uni-koeln.de; 4 Center of Physiology and Pathophysiology, Faculty of Medicine, University of Cologne, Robert-Koch-Str. 39, 50931 Cologne, Germany; 5 Translational Biogerontology, German Center for Neurodegenerative Diseases (Deutsches Zentrum für Neurodegenerative Erkrankungen, DZNE), Venusberg-Campus 1/99, 53127 Bonn, Germany; dan.ehninger@dzne.de; 6 German Center for Neurodegenerative Diseases (Deutsches Zentrum für Neurodegenerative Erkrankungen, DZNE), Venusberg-Campus 1/99, 53127 Bonn, Germany; 7 Center for Translational Medicine, Medical Faculty, University of Bonn, 53113 Bonn, Germany

**Keywords:** Alzheimer’s disease, APP, calcium channel, drugs, GABA, hippocampus, interneuron, oscillation, pharmacoepidemiology, pharmacotherapy, septum, theta, T-type

## Abstract

Voltage-gated Ca^2+^ channels (VGCCs) were reported to play a crucial role in neurotransmitter release, dendritic resonance phenomena and integration, and the regulation of gene expression. In the septohippocampal system, high- and low-voltage-activated (HVA, LVA) Ca^2+^ channels were shown to be involved in theta genesis, learning, and memory processes. In particular, HVA Ca_v_2.3 R-type and LVA Ca_v_3 T-type Ca^2+^ channels are expressed in the medial septum-diagonal band of Broca (MS-DBB), hippocampal interneurons, and pyramidal cells, and ablation of both channels was proven to severely modulate theta activity. Importantly, Ca_v_3 Ca^2+^ channels contribute to rebound burst firing in septal interneurons. Consequently, functional impairment of T-type Ca^2+^ channels, e.g., in null mutant mouse models, caused tonic disinhibition of the septohippocampal pathway and subsequent enhancement of hippocampal theta activity. In addition, impairment of GABA A/B receptor transcription, trafficking, and membrane translocation was observed within the septohippocampal system. Given the recent findings that amyloid precursor protein (APP) forms complexes with GABA B receptors (GBRs), it is hypothesized that T-type Ca^2+^ current reduction, decrease in GABA receptors, and APP destabilization generate complex functional interdependence that can constitute a sophisticated proamyloidogenic environment, which could be of potential relevance in the etiopathogenesis of Alzheimer’s disease (AD). The age-related downregulation of T-type Ca^2+^ channels in humans goes together with increased Aβ levels that could further inhibit T-type channels and aggravate the proamyloidogenic environment. The mechanistic model presented here sheds new light on recent reports about the potential risks of T-type Ca^2+^ channel blockers (CCBs) in dementia, as observed upon antiepileptic drug application in the elderly.

## 1. Preluding Remarks

This gap-bridging review integrates information from different scientific subfields, including molecular biology, electrophysiological, pharmacological, pharmacoepidemiological, and pharmacovigilance data. As a blueprint region for other brain areas, we start with a structural and functional description of the septohippocampal system and the mechanisms of theta oscillation subtypes in this region relevant for cognitive processes. Next, the functional implications of VGCCs in this system are elucidated, with a specific focus on Ca_v_3.x T-type VGCCs and the lessons learned from studies in Ca_v_3.x mutant mouse lines. The latter include alterations in GABAergic transmission and GABA receptor expression and recently described associations with APP degradation and AD pathophysiology. Given the functional interdependence of VGCCs, the GABAergic system, and APP processing, experimental blockers/enhancers of Ca_v_3.x channels, as well as licensed T-type modulators, are discussed based on preclinical and pharmacoepidemiological studies. In the final section, we provide a comprehensive mechanistic model proposal of how T-type VGCC block could potentially enhance a proamyloidogenic environment and why this feature warrants close pharmacovigilance observation of drugs with T-type blocking properties in the future.

## 2. Hippocampal Theta Oscillations: Structural and Functional Aspects and Relevance for Cognition and Memory

**Figure 1:**
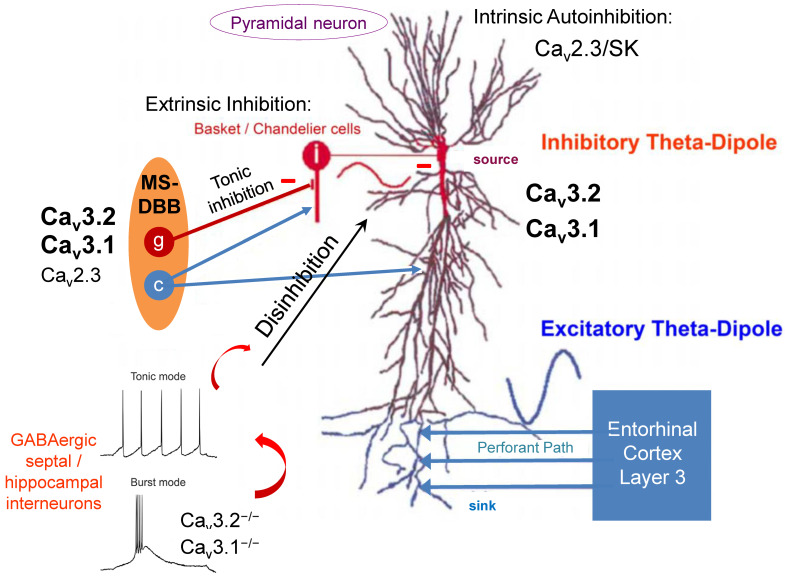
T-type VGCCs in hippocampal theta genesis. Septal GABAergic interneurons express both Ca_v_3.1 and Ca_v_3.2 VGCCs and project on hippocampal interneurons. Ablation of both T-type Ca^2+^ channel entities significantly impairs burst activity and favors the tonic mode of action in septal interneurons. The latter exert tonic inhibition of hippocampal GABAergic interneurons, resulting in disinhibition of hippocampal pyramidal neurons. Consequently, hippocampal type II theta oscillations are increased in Ca_v_3.1^−/−^ and Ca_v_3.2^−/−^ mice (MS-DBB, medial septum-diagonal band of Broca; the illustration of the septohippocampal pathway was partially modified from Buzsaki et al. (2002)). Note that other VGCCs such as HVA Ca_v_2.3 R-type Ca^2+^ channels are also expressed in the septohippocampal system and likely contribute to theta genesis. This image focuses on GABAergic transmission in the septohippocampal system. However, other transmitters such as ACh (see cholinergic (c) neurons in the MS) or glutamate also play an important role in septohippocampal rhythmicity and theta genesis.

Hippocampal theta oscillations are characterized by species-specific frequencies and are of high importance in various behavioral and cognitive processes, such as arousal, voluntary movement, attention, exploration, sensorimotor integration, learning, memory (including differential modulation of memory encoding and consolidation), and rapid eye movement (REM) sleep [1,2,3,4,5,6,7,8,9,10,11]. Here, we will pay specific attention to memory formation, in which the entorhinal cortex (EC) plays a crucial role, serving as an interface between the cortex and hippocampus. During active learning, principal neurons in layers II and III of the EC receive sensory inputs from the neocortex and project to the hippocampus for memory encoding [12]. This process activates the trisynaptic pathway from the EC to the dentate gyrus (DG), the CA3 area, and, ultimately, to the CA1-region (via Schaffer collaterals) as part of the encoding pathway [13]. Alternatively, there is a direct projection to the CA1 area using the temporoammonic pathway (EC–CA1), which is critical for memory consolidation [14]. Conversely, principal neurons, predominantly from layer V of the EC, receive direct hippocampal output and transfer information back to the cortex for memory consolidation [15]. Various interneuronal cell types are involved in modulating these processes [16]. For example, a negative-feedback mechanism for controlling the hippocampal output of information involves oriens lacunosum-moleculare interneurons in the CA1 region. For the generation of theta oscillations discussed here, other interneurons, such as Basket/Chandelier cells, are more critical (Figure 1). Chandelier cells comprise up to ~4% of CA1 hippocampal interneurons, whereas parvalbumin (PV)-positive Basket cells are estimated to make up ~14% of CA1 interneurons [16]. The major projecting target is the perisomatic region of postsynaptic principal neurons (Figure 1). The resultant phase relationship and relative magnitude of the perisomatic inhibitory and peripheral dendritic excitatory dipoles of hippocampal pyramidal neurons are hypothesized to generate theta (θ) waves in the CA1 region [17,18]. As elaborated below, voltage-gated Ca^2+^ channels (VGCCs) and the GABAergic system play key roles in the establishment and maintenance of theta oscillations in hippocampal pyramidal neurons [17,18]. Clearly, the hippocampus is structurally interconnected in a complex fashion, and those subregions directly engaged in theta genesis also receive innervation from numerous other brain areas. The latter contribute to the coding of sensory and motor information and can link the modulation of theta/alpha activity to behavioral states [6,19].

## 3. Atropine-Insensitive Type I and Atropine-Sensitive Type II Theta Activity in the Hippocampus

On the structural level, the medial septum-diagonal band of Broca (MS-DBB) and the hippocampus emerged as the core substrate to trigger and maintain complex theta oscillations [20,21,22,23,24,25]. Although still under investigation, it has been proposed that the MS-DBB initiates theta oscillations in the septohippocampal system. The related septal pacemaker-hippocampal follower model is widely acknowledged and based, i.a., on investigations by Hangya et al. (2009), which demonstrated that a subfraction of GABAergic medial septum (MS) neurons exerts pacemaker function and projects rhythmic output on hippocampal interneurons and pyramidal cells [26]. The exact anatomical position/origin of the theta oscillator has been discussed scientifically for long, including both intrahippocampal and extrahippocampal theories [27]. Notably, hippocampal theta operation is heterogeneous in nature. Given the dualistic theory of theta oscillations, one can distinguish atropine-insensitive type I theta from atropine-sensitive type II theta activity [17,18,28]. However, we are still lacking detailed information on the exact molecular/biochemical, in vitro, and in vivo electrophysiological and behavioral characteristics of these hippocampal theta oscillatory entities [29]. Atropine-insensitive type I theta activity seems to predominate during awakening, voluntary behavior, and movement and was proposed to be associated with metabotropic group I glutamate receptor activity, as well as with α-amino-3-hydroxy-5-methyl-4-isoxazolepropionic acid (AMPA) and N-methyl-D-aspartate (NMDA) receptor modulation [17,18,29,30,31]. In contrast, atropine-sensitive type II theta oscillations usually appear, i.a., during alert immobility and urethane-induced anesthesia [17,18,30,32,33]. Type II theta activity can be triggered by stimulation of muscarinic type 1/3 (M_1_/M_3_) G-protein-coupled receptors (GPCRs). The latter initiate the muscarinic signal transduction cascade, including G protein q/11 alpha subunit (Gα_q/11_), phospholipase C β_1/4_ (PLCβ_1/4_), inositol trisphosphate (InsP_3_), diacylglycerol (DAG), Ca^2+^, and protein kinase C (PKC) [28,34]. A number of downstream phenomena of this muscarinic cascade that are likely to be involved in type II theta genesis have been proposed in the literature. The latter include, i.e., suppression of a slowly activated K^+^ current (Is,_AHP_), a voltage- and time-dependent K^+^ current (I_M_), and a time- and voltage-independent leakage K^+^ current (K_leak_) [28,35,36]. In addition, the potentiation of a Ca^2+^-dependent, nonspecific cation current (I_CAT_) and a hyperpolarization and cyclic-nucleotide-gated current (I_h_) have also been favored [36,37]. Importantly, gene inactivation studies in mice affecting hippocampal PLCβ_1_ or septal PLCβ_4_ caused complete loss or significant attenuation of synchronized cholinogenic theta activity [34,38]. Overall, the potential downstream targets of muscarinic receptor activation, effective in neuronal excitability and theta genesis, seem to incorporate a sophisticated and well-organized armamentarium of ligand- and voltage-gated ion channels [36].

## 4. Voltage-Gated Ca^2+^ Channels in Type II Theta Genesis

Given the complex subcellular and cellular expression characteristics and functional involvement in dendritic resonance procedures, VGCCs display key elements in theta genesis, although the detailed mechanisms still need to be investigated [39,40]. For example, the high-voltage-activated (HVA) Ca_v_2.3 R-(resistant)-type Ca^2+^ channel represents one VGCC entity relevant for theta genesis and is known to be expressed in the hippocampus, particularly in GABAergic interneurons and the somatodendritic region of pyramidal cells [28,41,42,43,44,45,46]. In particular, Ca_v_2.3 inactivation impaired type II theta genesis and altered theta architecture in both spontaneous long-term EEG recordings and pharmacologically, i.e., urethane-induced theta oscillations [28]. Ca_v_2.3 VGCCs are of central importance in proictogenicity and neuronal (hyper)excitability [47,48,49]. Furthermore, hippocampal R-type Ca^2+^ currents are primarily mediated by Ca_v_2.3 α_1_-subunits [50,51,52] and enhanced by G_q/11_-coupled M_1_/M_3_ muscarinic acetylcholine receptor (mAChR) stimulation in both hippocampal neurons [53,54,55,56] and recombinant systems [57,58]. It has been proposed that stimulation of PKCδ, which belongs to the Ca^2+^-independent group II PKCs, is engaged in this process [59]. Tai et al. (2006) suggested earlier that M_1_/M_3_ mAChR activation via carbachol is capable of triggering hippocampal theta oscillations most likely via the G_q/11_, PLCβ_1_, and PKC-mediated activation of the Ca_v_2.3 R-type VGCC [57,60,61,62]. Furthermore, M_1_-M_5_ activation has been shown to differentially modulate T-type VGCC as well [63,64,65]. Importantly, theta oscillations can also be modulated by divalent heavy metal ions such as nickel (Ni^2+^), which exerts blocking effects on the Ca_v_2.3 Ca^2+^ channel [59,62]. Moreover, low micromolar concentrations of Ni^2+^ significantly impaired low-voltage-activated (LVA) T-type Ca^2+^ currents (see also Table 1). Based on these findings, it seemed unclear in which way LVA T-type Ca^2+^ channels could be functionally involved in hippocampal theta genesis.

Three different subtypes of T-type Ca^2+^ channels have been cloned, i.e., Ca_v_3.1 (α_1_G), Ca_v_3.2 (α_1_H), and Ca_v_3.3 (α_1_I) [45,66,67,68,69,70,71,72]. Electrophysiologically, T-type VGCCs are characterized by transient opening, fast voltage-dependent inactivation, and slow deactivation kinetics. Within the Ca_v_3 subfamily, Ca_v_3.3 Ca^2+^ channels have relatively slow inactivation kinetics, which makes this channel more prone to sustained burst firing than Ca_v_3.1 and Ca_v_3.2 VGCCs (IUPHAR/BPS, https://www.guidetopharmacology.org (accessed on 16 March 2022), see also Table 1). Importantly, T-type Ca^2+^ channels open upon small depolarization, and, as a result of overlapping steady-state inactivation and activation kinetics, they own the capability to generate a so-called “window” current at resting membrane potentials [73]. This phenomenon contributes to so-called bistability or excitatory destabilization in some neuronal cell types and is critical for the establishment of rhythmic oscillatory activity. The latter is involved in physiological processes such as sleep generation and maintenance, as well as hypnosis, sedation, and anesthesia [74]. Ca_v_3.x Ca^2+^ channels exhibit a broad expression pattern throughout the brain and mediate the modulation of intracellular Ca^2+^ homeostasis, neuronal excitability, and gene regulation [44,75,76,77,78]. In addition, LVA Ca^2+^ channels are engaged in action potential generation and propagation, Ca^2+^-dependent low-threshold currents (LTCs) and associated rhythmic (rebound) burst firing activities in some brain regions, synaptic plasticity, and neurotransmitter release [44,46,72,79]. In addition, T-type Ca^2+^ channels are relevant for numerous other physiological processes, such as sleep regulation, pain perception/processing, and body weight maintenance [66,67,75,80,81,82,83,84]. Furthermore, disruption of T-type VGCCs has been related to various neurological and neuropsychiatric diseases, including insomnia, epilepsy, Parkinson’s disease (PD), depression, schizophrenia, chronic pain syndromes, and sleep disorders [46,72,85,86,87,88,89,90,91,92,93,94,95,96]. However, detailed information available so far concerning the role of T-type Ca^2+^ channels in motor functions, affective, and cognitive processes is still limited. Disruption of T-type Ca^2+^ channel activity was shown to strongly modify the initiation and maintenance of long-term potentiation (LTP) in the hippocampal area, visual cortex, and cerebellum [97,98,99]. Given the fact that T-type Ca^2+^ channels interact with the neurotransmitter vesicle docking, fusion, and release machinery, it underlines the notion of their functional interdependence with synaptic transmission and LTP [100,101,102]. Furthermore, mutations of the human *CACNA1H* gene coding for Ca_v_3.2 have been associated with autism spectrum disorders (ASDs) [103].

**Table 1:** Biophysical, electrophysiological, and pharmacological characterization of LVA Ca_v_3.1–3.3 T-type VGCCs. Ten different pore-forming Ca_v_ α_1_-subunits have been cloned so far that are differentiated into three subfamilies, i.e., the HVA, dihydropyridine (DHP)-sensitive, L-type Ca_v_1.x channels, the HVA, DHP-insensitive Non-L-type Ca_v_2.x channels, and the LVA T-type Ca_v_3.x channels. Note that some HVA channels, such as Ca_v_1.3 and Ca_v_2.3, were also reported to exhibit moderate/mid-voltage activation thresholds and LVA characteristics under specific experimental and (patho)physiological settings. Each pore-forming α_1_-subunit is composed of four homologous domains (repeats I–IV), each of which contains six α-helical transmembrane segments (S1–S6). The pore-forming area is built up by the region between S5 and S6 of the four domains. Voltage-dependent gating of VGCCs is mediated by the voltage sensor, which is localized in the membrane spanning S4 segments and acts via highly conserved positive charges, predominantly arginine residues. Importantly, all Ca_v_ α_1_-subunit transcripts are subject to alternative splicing, which can significantly affect biochemical and electrophysiological properties. Various Ca_v_ α_1_-subunits coassemble with auxiliary subunits such as β_1–4_, α_2_δ_1–4_, and γ_1–8_ subunits. The auxiliary subunits are capable of modifying the biochemical, electrophysiological, and pharmacological properties of the VGCC complex, although this seems to be of less relevance in T-type Ca^2+^ channel physiology compared to the other subfamilies (Ca_v_1.x, Ca_v_2.x). This table summarizes a selection of genetic, biophysical, electrophysiological, and pharmacological properties of the Ca_v_3 subfamily, which is the focus of this review. In section (**A**), data on ion selectivity and conductance, gating inhibitors, and further Ca_v_3.x Ca^2+^ channel blockers are listed, including the negative decimal logarithm of the inhibitory concentration (50%) (pIC_50_) and the related holding potential(s) (mV). For details on the individual parameters, see: [68,95,104,105,106,107,108,109,110,111,112,113,114,115,116,117,118,119,120,121,122]. (**B**) Voltage dependence of activation and inactivation kinetics of Ca_v_3.x VGCCs in different species. For details on the individual parameters, see: [71,113,117,118,123,124,125,126,127,128]. Abbreviations: DRG, dorsal root ganglion; *Homo sapiens*; Mm, *Mus musculus*; RN, *Rattus norvegicus*; Sp, species; TCN, thalamocortical neuron; V0.5_act_, half-maximum activation voltage; V0.5_inact_, half-maximum inactivation voltage; 3β-OH, neurosteroid analog (3β,5β,17β)-3-hydroxyandrostane-17-carbonitrile. *, Electrophysiological data from Ca_v_3.x transfected HEK293 cells were obtained from different studies (see above).

A	Ca_v_3.1 (α_1_G) Genes: CACNA1G (Hs) Cacna1g (Mm), Cacna1g (Rn)	Ca_v_3.2 (α_1_H) Genes: CACNA1H (Hs) Cacna1h (Mm), Cacna1h (Rn)	Ca_v_3.3 (α_1_I) Genes: CACNA1I (Hs) Cacna1i (Mm), Cacna1i (Rn)
**Ion selectivity and** **conductance**	Sr^2+^ > Ca^2+^ > Ba^2+^ [7.3 pS] (Hs) Ba^2+^ = Sr^2+^ = Ca^2+^ (Rn)	Ca^2+^ [9.1 pS] = Ba^2+^ (Hs) Sr^2+^ = Ba^2+^ = Ca^2+^ (Rn)	Ca^2+^ [11.0 pS] (Hs) Sr^2+^ = Ba^2+^ = Ca^2+^ (Rn)
**Gating inhibitors** (pIC_50_, Holding voltage, Sp.)	**Kurtoxin** 7.3–7.8, −90.0 mV (Rn)	**Kurtoxin** 7.3–7.6, −90.0 mV (Rn)	**Kurtoxin** Kurtoxin not tested on Ca_v_3.3
**Channel blocker** (pIC_50_, Holding voltage, Sp.)	**Pimozide** 7.5, −100.0 mV (Rn)	**Pimozide** 7.3, −100.0 mV (Rn)	**Pimozide** 7.5, −100.0 mV (Hs)
	**Z944** 7.3, −80.0 mV (Hs)	**Z944** 6.8, −75.0 mV (Hs)	**Z944** 7.0, −75.0 mV (Hs)
	**TTA-P2** 7.0, −90.0 mV (Rn)	**TTA-P2** 7.0, −90.0 mV (Rn)	**TTA-P2** 7.0, −90.0 mV (Rn)
	**TTA-A2** 7.0, −75.0 mV (Hs)	**TTA-A2** 8.0, −75.0 mV (Hs)	**TTA-A2** 7.5, −75.0 mV (Hs)
	**ML218** 6.5, −90.0 mV (Hs)	**ML218** 6.5, −90.0 mV (Hs)	**ML218** 6.5, −90.0 mV (Hs)
	**Mibefradil** 6.0–6.6, −110.0–−100.0 mV (Hs)	**Mibefradil** 5.9–7.2, −110.0–−80.0 mV (Hs)	**Mibefradil** Hs: 5.8, −110.0 mV (Hs)
	**NNC 55-0396** 6.8–7, −70 mV (Hs)	**----**	**----**
	**(-)-(R)-efonidipine** 5.0–7.0, −100.0–−60.0 mV (Rn)	**----**	**----**
	**Anandamide** 5.4, −80.0 mV (Hs)	**Anandamide** 6.5, −80.0 mV (Hs)	**Anandamide** 6.0, −80.0 mV (Hs)
	**ABT-639** 5.0, −110.0 mV (Hs)	**ABT-639** 5.6, −110.0 mV (Hs)	**ABT-639** 5.0, −110.0 mV (Hs)
		**3β-OH** 5.5, --- (Rn)	**3β-OH** 5.7, --- (Rn)
	**Ni^2+^** 3.6–3.8, −90.0 mV (Rn)	**Ni^2+^** 4.9–5.2, −90.0 mV (Hs)	**Ni^2+^** 3.7–4.1, −90.0 mV (Rn)
**Channel activators**	**ST101** at 0.1 nM (Mm) sig. Ca^2+^ current increase	**----**	**----**
	**SAK3** 0.1–10 nM sig. Ca^2+^ current increase (Mm)	**SAK3** 0.1–10 nM no increase in Ca^2+^ current (Mm)	**SAK3** 0.1–10 nM sig. Ca^2+^ current increase (Mm)
**B**			
**Voltage dependence**			
	**TCN (Rn):**	**DRG (Rn):**	**----**
V0.5_act_	−63.0 mV	−47.4 mV	
τ_act_	2.0–8.0 ms	2.0–7.0 ms	
V0.5_inact_	−83.5 mV	−70.8 mV	
τ_inact_	20.0–50.0 ms	25.0–75.0 ms	
	HEK 293 (Hs) *:	HEK 293 (Hs) *:	HEK 293 (Hs) *:
V0.5_act_	−56.0–−46.0 mV	−59.9–−51.8 mV	−44.9–−41.8 mV
τ_act_	1.0–7.0 ms	1.6–8.4 ms	5.9–53.0 ms
V0.5_inact_	−78.0–−62.0 mV	−86.5–−81.7 mV	−72.0–−71.5 mV
τ_inact_	15.0–40.0 ms	12.9–32.6 ms	68.0–127.0 ms
	HEK 293 (Hs) *:	HEK 293 (Hs) *:	HEK 293 (Hs) *:
V0.5_act_	−51.2–−45.7 mV	−43.7 mV	−38.7–−37.2 mV
τ_act_	no values	1.8–9.9 ms	5.0–45.0 ms
V0.5_inact_	−80.6–−65.0 mV	−78.8 mV	−73.1–−71.0 mV
τ_inact_	11.1–21.8 ms	15.0–28.0 ms	66.0–111.0 ms
	HEK 293 (Hs) *:	HEK 293 (Hs) *:	HEK 293 (Hs) *:
V0.5_act_	−45.2 mV	−44.0–−38.6 mV	−40.6 mV
τ_act_	1.1–8.2 ms	2.0–10.0 ms	No values
V0.5_inact_	−76.9 mV	−56.6–−46.9 mV	−68.9 mV
τ_inact_	16.0–62.0 ms	20.0–120.0 ms	89.1–272.9 ms

Recently, Gangadharan et al. (2016) investigated theta activity in global Ca_v_3.1^−/−^ mice and mice with MS-specific inactivation of the Ca_v_3.1 gene, focusing on potential neural processes mediating exploratory behavior [129]. Selective Ca_v_3.1 knockdown in the MS augmented object exploration. In contrast, the global Ca_v_3.1 null mutant mice exhibited both improved object and open-field exploration [129]. Importantly, only type II hippocampal theta activity was augmented in the MS Ca_v_3.1 knockdown animals. In global Ca_v_3.1^−/−^ mice, however, both type I and type II hippocampal theta rhythms were enhanced. This specific phenomenon seemed to be related to a severe increase in excitability of septohippocampal GABAergic interneurons and a balance shift from the burst to the tonic firing mode [129]. LVA T-type Ca^2+^ channels, such as Ca_v_3.1, are known to trigger and generate low-threshold Ca^2+^ spikes (LTCSs) and burst activity (rebound burst firing) in various neuronal cell types [130,131,132,133]. Thus, ablation of Ca_v_3.1 VGCCs caused tonic inhibition of hippocampal GABAergic interneurons via tonic activation of projecting GABAergic interneurons in the MS (Figure 1). Consequently, perisomatic disinhibition of hippocampal pyramidal cells was speculated to increase theta activity in Ca_v_3.1 null mutant mice [129,134,135].

It should be noted that other Ca_v_3 T-type Ca^2+^ channels are also expressed in the septohippocampal system at higher levels, particularly Ca_v_3.2, which is coexpressed with Ca_v_3.1 VGCCs, in some structures involved in theta genesis even exceeding expression levels of Ca_v_3.1 [136]. Therefore, a more detailed look at the functional implications of Ca_v_3.2 VGCCs in theta genesis seems worthwhile.

In the brain, immunoreactivity for Ca_v_3.2 Ca^2+^ channels was generally more widely distributed than for Ca_v_3.1. Overall, intense Ca_v_3.2 immunoreactivity was detected in the hippocampus, cerebellum, septum, nucleus caudatus/putamen, and cortex, with moderate labeling in the thalamus, faint labeling in the midbrain nuclei, and no expression in the corpus callosum. In the hippocampus, immunoreactivity for Ca_v_3.2 was predominant in the stratum oriens and stratum radiatum of the CA1 and CA3 region and the stratum lucidum of the CA3 area [136]. In the dentate gyrus, immunoreactivity for Ca_v_3.2 was very prominent in the outer part of the stratum moleculare. Moderate labeling was observed in the inner 2/3 of the stratum moleculare of the dentate gyrus and weaker labeling also in the stratum lacunosum-moleculare of the CA1 and CA3 regions. Notably, the weakest intensity was detected in the stratum pyramidale of the CA1 and CA3 regions and the granule cell layer of the dentate gyrus [136]. On the subcellular hippocampal level, Ca_v_3.2 was detected in all dendritic subfields of the CA1 region. Ca_v_3.2 was observed in the extrasynaptic plasma membrane of dendritic spines and shafts, as well as in intracellular membranes [136]. Importantly, dendritic spines in the stratum oriens and stratum radiatum exhibited strong labeling for Ca_v_3.2, but this intense labeling decreased severely in the stratum lacunosum-moleculare. Apart from pyramidal neurons, Ca_v_3.2 VGCCs were also observed in dendritic shafts of interneurons. Presynaptically, Ca_v_3.2 was observed in axon terminals forming asymmetrical synapses with dendritic spines throughout all dendritic layers [136]. Overall, the expression pattern of Ca_v_3.2 VGCCs allows for sophisticated neuronal synaptic, dendritic, and somatic integration and complex involvement in theta genesis. In this context, previous studies demonstrated that Ca_v_3.2 Ca^2+^ channels are of central relevance for hippocampal LTP, cued-context fear conditioning, and passive avoidance tasks [98]. Furthermore, deletion of Ca_v_3.2 was demonstrated to facilitate anxiety-related behavior, impair learning and memory formation, and result in reduced sensitivity to psychostimulants [137].

Recently, we investigated the role of Ca_v_3.2 T-type Ca^2+^ channels in initiation, maintenance, and modulation of hippocampal theta oscillations and the immanent molecular/biochemical and in vivo electrophysiological mechanisms. We showed that Ca_v_3.2^−/−^ mice display enhanced type II theta activity in spontaneous 24 h long-term EEG recordings [138]. Importantly, the increase in theta/alpha relative EEG power was most prominent during the inactive state of the light cycle, as well as the dark cycle. This inactive state is typically associated with stages of alert immobility, a physiological status characterized by hippocampal type II theta activity. Therefore, alterations in theta activity observed in Ca_v_3.2^−/−^ mice are likely to be based on the atropine-sensitive subtype II theta entity. These results were further confirmed by urethane injection studies, which revealed a significant increase in type II theta activity in Ca_v_3.2-deficient mice compared to controls [138]. Pharmacodynamically, urethane exhibits a multitarget character, exerting both stimulatory and inhibitory action on various voltage- and ligand-gated ion channels. Although urethane serves as an agonist on muscarinic and nicotinic AChR, GABA A receptors, and glycine receptors, it exerts antagonistic effects on NMDA and AMPA receptors as well [139,140]. It is important to stress that both Ca_v_3.2^+/+^ and Ca_v_3.2^−/−^ mice displayed typical circadian activity profiles. No significant differences in motor activity were detected between both genotypes, indicating that alterations in the hippocampal theta/alpha band were not based on changes in locomotion [138].

## 5. T-Type Ca^2+^ Channel Inactivation and Implications for the GABAergic System

**Figure 2:**
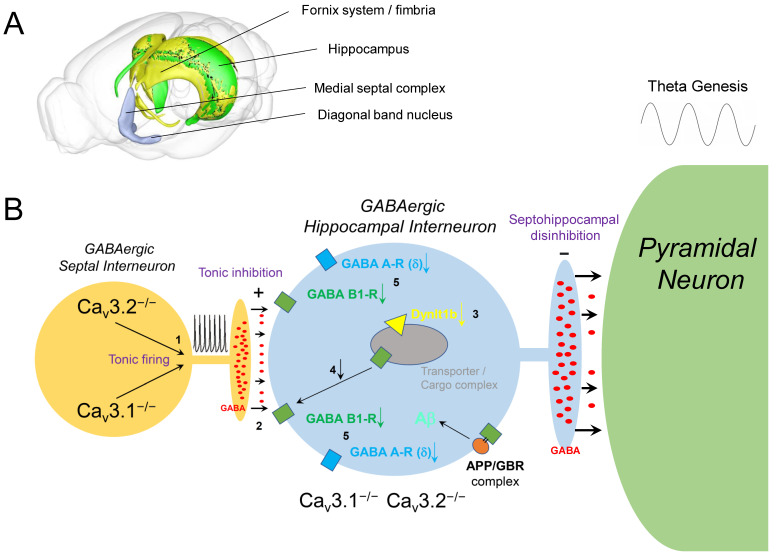
Altered GABAergic physiology in the septohippocampal system upon Ca_v_3 VGCC ablation. (**A**) This 3D illustration of the mouse brain including the septohippocampal system/pathway was generated using the “Scalable Brain Atlas” [151]. Blue, medial septal complex, diagonal band of Broca; green, hippocampus; yellow, fornix system/fimbria. (**B**) Increased GABA release from septal interneurons upon tonic firing (1) is supposed to enhance GABA release into the synaptic cleft (2) and decrease GABA receptor density in hippocampal interneurons. This hypothesis is supported by a reduction of Dynlt1b transcripts (3). Dynein-containing cellular transportomes were reported to mediate GABA receptor transfer and integration into the sub- and postsynaptic membrane (4). In addition, Ca_v_3.2 ablation was associated with a reduction of GABA A receptor δ subunit and GABA B1 receptor subunit transcripts (5). Importantly, δ subunit containing GABA A receptors are also localized extrasynaptically and are known to mediate tonic inhibition. As APP forms complexes with GBRs, it is speculated that APP/GBR microassemblies are destabilized in Ca_v_3.2-deficient mice as well. In consequence, T-type Ca^2+^ channel/current reduction with age or by pharmacological interference could generate a proamyloidogenic environment relevant in the etiopathogenesis of AD and its progression.

Previously, detailed transcriptome studies from the hippocampus of both Ca_v_3.2^+/+^ and Ca_v_3.2^−/−^ mice were performed [141]. Subsequent qPCR analysis of transcriptome gene candidates elicited a significant reduction in dynein light chain Tctex-type 1 (Dynlt1b) in Ca_v_3.2^−/−^ mice [138]. The latter serves as part of the GABA receptor transportome complex, which mediates the translocation of GABA receptors to the subsynaptic or extrasynaptic membrane areas [142,143,144]. This finding pointed to alterations of GABAergic transmission in the septohippocampal system of Ca_v_3.2-deficient mice (Figure 2). Arshaad et al. (2021) further analyzed the transcript levels of GABA A and GABA B receptors (GBRs) in the hippocampus. In Ca_v_3.2 null mutant mice, GABA A receptor δ subunits and GABA B1 receptor subunits exhibited a significant reduction in transcript levels [138]. These results strongly support our GABA-based hypothesis of enhanced theta/alpha activity in Ca_v_3.2^−/−^ mice: Within the CNS, GABA A receptor-mediated inhibition was proven to occur by fast synaptic neurotransmission and sustained tonic inhibition [145]. Studies in dentate gyrus granule cells and thalamic neurons further demonstrated that extrasynaptically localized GABA A receptors that contain, e.g., δ-subunits, mediate tonic current that is critical for excitatory phenomena in neurons/interneurons in response to circumjacent GABA concentrations [146,147,148]. On the other hand, slow postsynaptic inhibition observed in dendritic spines can be mediated by GABA B1 subunit containing receptors [149,150].

**Figure 3:**
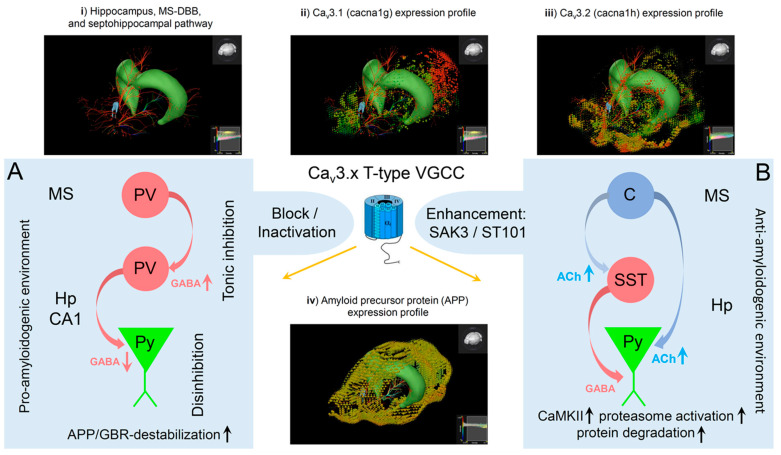
Ca_v_3.x VGCC activation and inhibition and the potential consequences for anti- and proamyloidogenic effects. The 3D illustrations present (i) the hippocampus, the MS-DBB, and the connecting fibers; (ii) the 3D projection of Ca_v_3.1 (α_1_G) transcript data/expression; (iii) the 3D projection of Ca_v_3.2 (α_1_H) transcript data/expression; and (iv) the 3D projection of the APP transcript/expression profile. Transcript data points are displayed in sagittal sections for the left brain. Three-dimensional (3D) illustrations and integration of transcript data were done using Allen Brain Mouse Atlas - Brain Explorer^®^ 2 [286]. Note that no 3D representation of Ca_v_3.3 (α_1_I) expression is presented, as transcript data were not available using Brain Explorer^®^ 2. (**A**) Ca_v_3.x VGCCs can be blocked by various antagonists with different specificities. As outlined in our proposed model, inhibition of T-type VGCCs can favor tonic firing in parvalbumin (PV)-positive interneurons in the medial septum (MS). Subsequently, increased GABAergic projection on PV-positive hippocampal GABAergic interneurons results in functional disinhibition of hippocampal pyramidal neurons (Py). A reduction in GBR expression and/or cell surface expression could lead to destabilization of the APP/GBR complex as described previously. In total, inhibition of Ca_v_3.x T-type VGCCs might constitute a proamyloidogenic environment. (**B**) Activation of T-type VGCCs, e.g., via ST101 or SAK3, was shown to enhance ACh release from septal cholinergic neurons. The latter can project on somatostatin (SST)-positive GABAergic interneurons or pyramidal neurons (Py) in the hippocampus. It has been suggested that Ca_v_3.x mediated enhancement of ACh release might trigger CaMKII- and proteasome activation with subsequent enhancement of protein degradation. Thus, activation of T-type Ca^2+^ channels might generate an antiamyloidogenic environment. In summary, inhibition of Ca_v_3.x is suggested to trigger Aβ synthesis and plaque formation via destabilization of APP/GBRs assemblies and reduced CaMKII/proteasome activation. In contrast, stimulation of Ca_v_3.x is likely to stabilize APP/GBR microcomplexes and to enhance CaMKII/proteasome-mediated Aβ/plaque degradation.

Importantly, results from microarray analysis or qPCR studies did not favor the interference of potential compensatory transcriptional alterations of other T-type Ca^2+^ channels, i.e., Ca_v_3.1 and Ca_v_3.3 in the hippocampus of Ca_v_3.2^−/−^ mice [138]. In addition, transcriptome analysis did not reveal alterations in other voltage- and ligand-gated ion channels, despite the GABAergic system [141]. However, electrophysiological changes in the latter systems, e.g., in voltage-gated Na^+^ channels/currents, cannot be excluded, as ion channel regulation also depends on alternative splicing, post-translational modification, subunit composition, and further complex regulation via signaling cascades. Thus, the theta/alpha alterations observed in Ca_v_3.2 null mutant mice seemed to be solely attributable to Ca_v_3.2 ablation itself [138]. In summary, transcriptome data and qPCR findings suggest that both subsynaptic/postsynaptic and extrasynaptic GABA receptor transcripts are diminished upon tonic inhibition of GABAergic hippocampal interneurons in Ca_v_3.2^−/−^ mice and that decreased plasma membrane density of GABA receptors is based on malfunction/insufficiency of a dynein/GABA receptor containing transportome complex and its related trafficking (Figure 2 and Figure 3).

## 6. GABAergic Neurotransmission and APP/Aβ Processing in Alzheimer’s Disease

The amyloid precursor protein (APP) serves as the key polypeptide originator from which all amyloid-beta (Aβ) peptide variants are finally derived from upon sequential proteolytic processing via β-site APP cleaving enzyme (BACE) and γ-secretases [152,153,154]. A detailed elaboration of the etiopathogenesis of AD has been given in various excellent reviews [155,156,157,158,159,160] and will not be further addressed here. Instead, this review focuses on the modulation of APP/Aβ processing in the context of a proamyloidogenic environment. Notably, APP exerts important physiological functions in the mammalian brain which are specifically related to the modulation of synaptic transmission and neuronal survival [161,162,163]. This phenomenon is based on the interaction of APP/Aβ with different voltage- and ligand-gated ion channel subgroups, two of which will be addressed in this review, i.e., the GABAergic system and VGCCs.

Evidence has been provided that alterations in the GABAergic system, e.g., downregulation of presynaptic GBRs, can occur in response to neuronal activity [164,165,166] and in different diseases, such as the Fragile-X syndrome [167], epilepsies [168], Parkinson’s disease [169], and AD [170,171,172,173]. This GBR downregulation was often interpreted as a consequence or secondary effect of the disease, e.g., based on excessive GABA release by reactive astrocytes and subsequent enhanced GBR activation [174,175]. Similarly, altered axonal transport, which serves as a pathological feature of AD and is related to increased Aβ levels [176,177], was also shown to diminish GABA B receptor 1a/2 (GBR1a/2) expression on glutamatergic synaptic terminals and to enhance NMDA-receptor-mediated GBR degradation [164,178]. Conversely, NMDA receptor inhibition prevented GBR degradation and led to stabilization of APP/GBR1a complexes [164,165,178]. Notably, it has been speculated that downregulation of GBR [171,172] may not only increase Aβ levels but also enhance excitotoxic effects and thus contribute to seizure activity and memory impairment in AD [179]. In this context, Memantine, a noncompetitive NMDA receptor antagonist used in the treatment of AD, was suggested to stabilize APP in the cell membrane and reduce Aβ processing [180]. In particular, it was hypothesized that Memantine stabilizes APP in the cell membrane by inhibition of NMDA-receptor-mediated GBR internalization [164,165,178]. In addition, GBR antagonists were also shown to stabilize GBRs at the cell surface by precluding GBR degradation [181]. Thus, it is not astonishing that GBR antagonists are also promising targets in drug research and development as potential novel therapeutics in AD, as they might promote proper synaptic processing relevant for cognition, learning, and memory [174,181].

Studies, particularly in the last decade, have given a detailed insight into the functional interplay between APP/Aβ and GABA neurotransmission in two ways: (i) APP seems to modulate KCC2/SLC12A5, a neuron-specific K^+^/Cl^−^ cotransporter. This transporter significantly contributes to the maintenance of the intracellular neuronal Cl^−^ concentration and the related reversal potential and is thus essential for postsynaptic inhibitory processes mediated by ionotropic GABA A receptors [154]. (ii) In addition, APP interacts with the sushi domain of metabotropic GABA B receptor 1a (GBR1a). Most neurons in the brain express GBR1a. Within this complex, APP is cotransported with GBR dimers including GBR1a to the axonal presynaptic plasma membrane. Interestingly, secreted APP (sAPP) generated by secretase cleavage also seems to interfere with GBR1a and modulate the presynaptic vesicle release machinery [154]. In general, GBRs are known as key regulators of synaptic release [182,183]. Presynaptically, GBRs impair the liberation of various neurotransmitters, while postsynaptic GBRs generate hyperpolarizing inhibitory K^+^ currents and impede neuronal activity [154,184]. On the cellular level, the heterodimeric GBR1a/2 and GBR1b/2 assemblies accumulate at excitatory terminals and in the somatodendritic compartment, respectively [150,182,184,185,186]. It has been demonstrated that the selective genetic ablation of APP significantly impaired GBR-related presynaptic inhibition and axonal GBR expression [187]. Interestingly, further proteomic and functional analyses revealed that APP associates with other components, e.g., c-Jun N-terminal kinase-interacting protein (JIP) and calsyntenin, that bridge the functional and structural gap between the APP/GBR complex to cargo vesicles mediating axonal trafficking [187]. Additionally, APP was reported to link cargo vesicles via adaptor molecules to axonal kinesin-1 motor proteins [188,189,190]. Importantly, APP/GBR1a complexes do not only traffic anterogradely in axons as outlined above but additionally effectuate retrograde trafficking of APP/GBR1a complexes, probably mediated by dynein motors. Consequently, APP/GBR1a microassemblies were found in dendritic shafts as well [187,189]. This is not astonishing per se, as axonal proteins are not restricted to axons since both the Golgi apparatus and the endoplasmic reticulum (ER) extend into dendrites. However, it cannot be excluded that APP/GBR1a complexes are partially internalized in axons and transcytosed to the dendrites, as it has been proposed for APP [191,192]. The most striking phenomenon, however, is that GBRs stabilize APP in complexes at the cell surface and reduce the proteolysis of APP to Aβ, the latter serving as the critical step in the formation of senile plaques in AD patients [152,153,187,193]. Dinamarca et al. (2019) concluded that APP/GBR complex formation provides a sophisticated linkage between presynaptic and dendritic GBR trafficking and Aβ formation and that dysfunction in axonal trafficking and reduced GBR expression can be related to AD based on increased Aβ formation [187]. The latter is related to the fact that APP/GBR1a microassemblies limit the availability of APP for endosomal processing to Aβ under physiological conditions [187]. In addition to its protective role, GBR1a also keeps APP out of dendritic spines that are highly enriched with recycling endosomes [194]. Furthermore, APP links GBR1a/2 to vesicular trafficking and, upon deletion, mediates a significant impairment of GBR-mediated inhibition of glutamate release [187].

It is not astonishing that GABA receptors have been linked to the etiopathogenesis of numerous human traits and diseases, e.g., encephalitis, AD, PD, Rett syndrome, epileptic encephalopathy, generalized epilepsy, brain size alterations, pain, bipolar disorder, major depressive disorder, schizophrenia, migraine, glioblastoma multiforme, type 2 diabetes, and gastrointestinal tumors [195].

The functional implications of both GBR and APP outlined above were demonstrated in various studies using GBR1a and APP null mutant mice. Given the structural APP/GBR coassembly and functional interaction of both partners, the mutant models exert several phenotypical/symptomatic overlaps, such as impaired GBR-related presynaptic inhibition [150], enhanced seizure susceptibility [153,182], negative effects on LTP [150,153,196], cognitive impairment [150,182,196], dysregulated oscillatory network activity [197,198,199], and altered circadian locomotion [182,196]. Accordingly, cultured hippocampal neurons from GBR1a null mutant mice displayed a ~40% increase in released Aβ levels compared to control mice [187].

Obviously, there is a complex functional interdependence between GBRs and APP that seems to play a substantial role in the etiopathogenesis of AD and is capable of generating a complex proamyloidogenic environment. A central question remains: which factors could positively or negatively affect the stability of GBR/APP complexes, modulate Aβ production, and contribute to a proamyloidogenic or antiamyloidogenic environment? As outlined above, recent findings suggest that VGCCs, e.g., in the septohippocampal system, affect GABAergic transmission and GABA receptor trafficking. Therefore, the potential role of VGCCs in this context will be elucidated in the next section.

## 7. Functional Interdependence of VGCCs and APP/Aβ Processing

Although numerous cellular targets of Aβ have been reported in the past, the present review focuses on its functional interdependence with VGCCs. An indirect mechanism of action on VGCCs via modulation of membrane potentials and Ca^2+^ homeostasis is related to the inhibitory effect of Aβ on K^+^-channels, and recent findings suggested that voltage-gated K^+^ channels could be involved in Aβ-mediated neurodegenerative processes [200]. In particular, a transient A-type K^+^ current, with a complex spatial distribution pattern with increased plasma density from soma to dendrites in hippocampal CA1 pyramidal neurons, was proven to contribute to dendritic membrane excitability [200]. The persistent inhibition of the A-type K^+^ current due to Aβ accumulation in the dendritic arbor was suggested to mediate a sustained increase in dendritic Ca^2+^ levels and disturb Ca^2+^ homeostasis [200]. Notably, this mechanism may act not only on hippocampal pyramidal neurons but on other cellular components of the MS-DBB as well. Here, depolarization due to K^+^-channel inhibition and initially increased Ca^2+^-influx/Ca^2+^ dyshomeostasis could finally inactivate LVA VGCCs and lead to a shift from the (rebound) burst firing mode to the tonic mode as outlined above. Similar to the downregulation or genetic ablation of an LVA Ca_v_3 Ca^2+^ channel [129,138,201], these constellations would again favor tonic disinhibition in the septohippocampal pathway [129,138].

Recently, Gavello et al. (2018) analyzed the effects of Aβ42 on the Ca^2+^-dependent excitability profile of hippocampal neurons. Experiments were carried out on cultured hippocampal networks and revealed that Aβ42 differently modulates ryanodine receptors (RyRs), NMDA receptors (NMDARs), and VGCCs with enhanced Ca^2+^ release via RyRs and inhibition of Ca^2+^ influx via NMDARs and VGCCs [202]. In total, Aβ caused an increase in cytosolic Ca^2+^ concentration, which resulted in an activation of big conductance Ca^2+^-activated K^+^ channels (BK channels) and inhibition of hippocampal network firing [202]. Obviously, increased internal Ca^2+^ levels upon Aβ exposure seem to be a common feature in all experiments and are based on various ion channel entities. The Aβ effects on different Ca^2+^ channels, however, are more sophisticated, partially opposing, and may also be affected by the experimental settings. They will be elaborated and discussed in the following, starting with the functional interdependence between Aβ and VGCCs. Kim and Rhim (2011) demonstrated that Aβ25–35 acutely and chronically upregulates the HVA L-type Ca_v_1.3 VGCC in the rat hippocampus and HEK293 cells [203]. These findings suggested that Ca_v_1.3, along with Ca_v_1.2, another HVA VGCC, plays a potential role in the pathogenesis of AD [203]. Interestingly, Daschil et al. (2013) elaborated that the formation of Aβ plaques in AD mouse models is associated with Ca_v_1.2 Ca^2+^ channel expression also in reactive astrocytes [204]. Early in 2004, Bobich et al. (2004) demonstrated that incubation of nerve endings with a physiological concentration of Aβ1–42 activates Ca_v_2.2 (N-Type) VGCCs and acutely increases glutamate and noradrenaline release [205]. Later, Hermann et al. (2013) illustrated that synthetic Aβ oligomers (Aβ1–42 globulomers) modulate/activate presynaptic Ca^2+^ currents, and the authors concluded that Aβ-induced synaptic deficits could be avoided by Ca^2+^ channel blockers (CCBs) [206]. It has been suggested that Aβ oligomers directly compromise synaptic function, thereby causing cognitive deficits in AD. The synthetic Aβ oligomers were shown to directly modulate Ca_v_2.1 P/Q-type Ca^2+^ channels, possibly triggering excitotoxic cascades and subsequent synaptic decline [206]. On the electrophysiological level, Aβ globulomers shifted the half-activation voltage of Ca_v_2.1 P/Q-type and Ca_v_2.2 N-type Ca^2+^ channels to more hyperpolarized values. Interestingly, application of nonaggregated Aβ peptides exhibited no effect. Specific blockage of Ca_v_2.1 P/Q-type or Ca_v_2.2 N-type Ca^2+^ channels with peptide toxins fully reversed Aβ globulomer-induced impairment in glutamatergic neurotransmission [206]. However, inhibition of L-type VGCCs was not capable of reversing this deficit. Hermann et al. (2013) further demonstrated that Aβ globulomers directly modulate recombinant Ca_v_2.1 P/Q-type and Ca_v_2.2 N-type VGCCs in HEK293 cells. Blockage of these presynaptic Ca^2+^ channels with both state-dependent and state-independent modulators was capable of reversing Aβ-induced functional deficits in synaptic transmission [206]. These findings suggested that presynaptic CCBs may constitute a therapeutic strategy for the treatment of AD in the future. In contrast to the above findings, Sadleir et al. (2021) found that pregabalin, which binds to the auxiliary α_2_δ subunit of Ca_v_2.1 P/Q-type VGCCs, had no impact on amyloid pathology in the 5XFAD mouse model. Consequently, the authors concluded that HVA Ca^2+^ channel modulation does not seem to influence amyloid pathology [207].

Interestingly, Ishii et al. (2019) reported that in young Tg2576 transgenic mice overexpressing mutated APP, Aβ results in dysfunction in neuropeptide Y (NPY)-expressing hypothalamic arcuate neurons prior to plaque formation [208]. In this setting, the dihydropyridine (DHP) nimodipine was shown to hyperpolarize the neural membrane potential, reduce spontaneous activity, and decrease intracellular Ca^2+^ concentrations in arcuate NPY neurons from Tg2576 brain slices. In addition, there was a shift from high- to low-voltage-activated Ca_v_1.x L-type Ca^2+^ currents, causing an increased Ca^2+^ influx closer to the resting membrane potential, an effect recapitulated by Aβ1–42 and reversed upon nimodipine application [208]. The data also suggested that dysregulation of intracellular Ca^2+^ is only reversible during the early stages of Aβ pathology. Concisely, these findings provided evidence for a key role of low-threshold activated Ca_v_1.x L-type Ca^2+^ channels in Aβ-mediated neuronal dysfunction.

It is important to note that other VGCCs, i.e., LVA Ca_v_3.1 VGCCs, are also affected by Aβ. Notably, Aβ was reported to block T-type Ca^2+^ channels and cause blockage of the new synaptic assembly via Nogo receptors [209]. In the presence of Aβ, neurons were not able to form new synapses, causing significant impairment of learning in vivo. Zhao et al. (2017) also demonstrated that the Nogo receptor family (NgR1–3) serves as Aβ receptors and mediates inhibition of synaptic assembly, plasticity, and, finally, learning. Furthermore, these processes were associated with inhibition of Ca_v_3 T-type VGCCs. Thus, Aβ exerts dysregulation of Ca^2+^ homeostasis and impairment of synaptic physiology via two modes of action: (i) direct antagonistic effects on T-type Ca^2+^ channels and (ii) Aβ-NgR mediated signaling [209]. These findings by Zhao et al. (2017) are striking, as the accumulation of Aβ and blockage of T-type Ca^2+^ channels would mimic the conditions observed in Ca_v_3.1 and Ca_v_3.2 knockout mice outlined above and further promote the destabilization of APP/GBR complexes in terms of a vicious cycle.

Importantly, APP directly interacts with VGCCs as well. Yang et al. (2009) reported the critical role of APP in the regulation of HVA Ca_v_1.x L-type Ca^2+^ channels in GABAergic inhibitory neurons in both the hippocampus and striatum. Interestingly, APP deletion in mice led to an increase in Ca_v_1.2 VGCCs. The upregulated Ca_v_1.2 levels caused a reduction in GABAergic paired-pulse inhibition (reflecting GABA A receptor-mediated inhibition of principal neurons through local interneurons) and an increase in GABAergic post-tetanic potentiation in both hippocampal and striatal neurons. The latter indicates that APP modulates synaptic properties of GABAergic neurons via regulation of Ca_v_1.2 Ca^2+^ channels [210]. The authors further suggested that APP physically interferes with Ca_v_1.2 channels. In consequence, loss of APP could lead to an inappropriate accumulation and aberrant activity of Ca_v_1.2 VGCCs [210].

In addition, APP was shown to regulate depolarization-induced Ca^2+^-mediated synaptic signaling in brain slices via modification of trafficking to synapses and promotion of their formation [211]. On the synaptic level, APP interacts with synaptic proteins engaged in vesicle exocytosis and modulates Ca^2+^ channel function. In the absence of APP, decreased pCaMKII and pERK levels were also observed. This decrement was susceptible to the inhibition of Ca_v_2.1 P/Q- and Ca_v_2.2 N-type VGCCs by ω-conotoxin GVIA and ω-conotoxin MVIIC, respectively. However, it turned out to be insensitive to inhibition of L-type VGCCs by the DHP nifedipine [211]. These findings illustrate that APP also regulates synaptic-activity-mediated neuronal signaling by affecting P/Q- and N-type VGCCs. Interestingly, in specific clinical settings, e.g., acute hypoxia, selective expression of the secreted extracellular fragment sAPPα or pharmacological blockage of L-type VGCCs channels increased neuronal resistance [212].

## 8. VGCCs, GABAergic Neurotransmission, and APP Processing-Lessons Learned from Animal Models

Studies in APP/PS1 AD mice revealed changes in GABA synthesis and transport. Further investigations in the hippocampus from these mice exhibited a reduction in GBR receptor subunits on both the mRNA and protein levels (Salazar et al., 2021). Interestingly, these alterations in GABA receptor expression parallel those observed in Ca_v_3.2^−/−^ mice [138]. Notably, four-month-old APP/PS1 mice did not exhibit deficits in spatial learning and memory or changes in GABA signaling compared to controls. Within six months, however, significant alterations in GABA-associated targets, e.g., GBR, were detected that coincided with spatial learning deficits [213]. These findings illustrate that the APP/PS1 AD mouse model displays altered GABAergic signaling and consistent AD-related deficits [213]. The question arises if there is any indication that VGCCs contribute to this GABA phenotype in APP/PS1 mutant mice.

Yang et al. (2019) investigated molecular alterations in early-stage (seven months old) and late-stage (18 months old) APP/PS1 AD mice [214]. Analysis of differentially expressed genes in the hippocampus revealed significant upregulation of transcripts of 14 different Ca^2+^ channel subtypes in aged mice [214]. The latter included HVA VGCC, i.e., Ca_v_1.2 (α_1_C, Cacna1c), Ca_v_1.3 (α_1_D, Cacna1d), Ca_v_1.4 (α_1_F, Cacna1f), Ca_v_2.1 (α_1_A, Cacna1a), Ca_v_2.2 (α_1_B, Cacna1b), and Ca_v_2.3 (α_1_E, Cacna1e). These changes in Ca^2+^ channel genes turned out to be the prominent features in aged APP/PS1 mice [214] and once again point to a shift in the HVA-to-LVA Ca^2+^ channel ratio, which could trigger tonic inhibition in septal neurons and disinhibition in the septohippocampal pathway. Given the model presented in this review (Figure 2 and Figure 3), these changes in network activity can explain the reduced GBR expression and APP/GBR destabilization and might contribute to the AD symptoms in APP/PS1 mice.

The most striking results confirming our model were published by Rice et al. (2014). The authors demonstrated that age-dependent downregulation of the Ca_v_3.1 T-type VGCCs indeed served as a mediator of Aβ production [201]. It is well-known that age-related dysregulation in Ca^2+^ homeostasis occurs before and throughout the course of AD and that Ca^2+^ dyshomeostasis contributes to altered processing of APP, facilitation of Aβ production, and thus AD pathogenesis [215,216,217,218]. Rice et al. (2014) proved that downregulation of LVA Ca^2+^ current in murine Neuro 2a (N2a) cells and the 3xTg-AD mouse model by pharmacological inhibition via NNC-55-0396 (a highly selective T-type CCB) dramatically facilitated Aβ production. Consequently, APP expressing HEK-269 cells overexpressing Ca_v_3.1 Ca^2+^ channels exhibited contrary effects, i.e., nonamyloidogenic processing with an almost 3-fold increase in sAPP-α. Furthermore, τ phosphorylation remained unchanged [201].

Importantly, Rice et al. (2014) also analyzed human microarray data sets from young (20–59 a old)/aged (74–95 a old) nondemented and demented individuals revealing a consistent and dramatic age-related impairment of CACNA1G gene expression, which encodes for the human Ca_v_3.1 T-type VGCC. The tissue for microarray data was obtained from different brain regions, including the entorhinal cortex (EC), the hippocampus (HC), the posterior cingulate gyrus (PCG), and the superior frontal gyrus (SFG). The mRNA expression levels of Ca_v_3.1 VGCCs exhibited an age-related reduction of 41%, 49%, 45%, and 46% in the EC, HC, PCG, and SFG, respectively [201]. Strikingly, the additional decrease in Ca_v_3.1 expression in AD patients characterizes Ca_v_3.1 (CACNA1G) as a critical factor of Ca^2+^ homeostasis and facilitates cognitive impairment. Overall, there is an age-related reduction of the Ca_v_3.1 T-type VGCC at the mRNA and protein level in both humans and mice. The latter exacerbates in the presence of AD and is associated with a rapid increase in Aβ levels [201]. Consequently, the age-related decrease in Ca_v_3.1 expression may contribute to a proamyloidogenic environment in the aging brain. Moreover, T-type Ca^2+^ channels may represent a novel opportunity to interfere with the etiopathogenesis and progression of AD and serve as a novel target in its pharmacotherapeutic intervention [201].

It is important to consider that Rice et al. (2014) also reported a significant, but lower, age-related decrease for (i) Ca_v_1.3 and Ca_v_2.3 in the EC, (ii) Ca_v_2.1, Ca_v_2.2 and Ca_v_1.3 in the PCG, and (iii) Ca_v_2.2, Ca_v_1.3, Ca_v_2.3 and Ca_v_3.3 in the SFG, i.e., from young to aged nondemented individuals [201]. As outlined above, some of these channels also contribute to the neuronal balancing of tonic and (rebound) burst firing that seems critical in GABA regulation and GBR/APP stabilization.

## 9. Implications for Pharmacoepidemiology and Pharmacotherapy

Numerous studies suggest that Ca^2+^ dysregulation within the cytosol, the internal stores (ER), and other cell organelles (mitochondria) plays an essential role in AD and causes additional AD-related abnormalities, e.g., inflammatory processes, elevated levels of reactive oxygen species (ROS), impaired autophagy, neurodegeneration, synaptic dysfunction, and cognitive decline. Pharmacotherapeutic approaches to restore proper Ca^2+^ homeostasis in AD are thus judged to be promising based on studies in preclinical models [219,220]. Several studies investigated the effects of VGCC blockers in AD. Anekonda et al. (2011) suggested that L-type VGCC blockade, e.g., with isradipine, could serve as a therapeutic strategy for AD [221,222]. Yagami et al. (2012) systematically reviewed the role of L-type VGCCs as therapeutic targets for neurodegenerative diseases with a specific focus on biochemical characterizations, physiological functions, pathological roles, and pharmacological applications [223]. As outlined above, presynaptic Ca_v_2.1 P/Q-, Ca_v_2.2 N-, and Ca_v_2.3 R-type VGCCs trigger neurotransmitter release, and Ca_v_3.x T-type VGCCs support neuronal rhythmic burst firing. Aβ, a causative factor and pathological hallmark for AD, potentiates the influx of Ca^2+^ into neurons via L-type VGCCs. Thus, L-type VGCC blockers were suggested to prevent neurons from undergoing Aβ-induced apoptosis [223]. Goodison et al. (2012) also investigated CCBs in AD. Based on the assumption that Aβ peptides result in an increase in intracellular Ca^2+^ via VGCCs, Goodison et al. (2012) also suggested that CCB action in the brain could potentially delay the onset and progression of AD [224]. Cataldi et al. (2013) analyzed the changing landscape of VGCCs in neurodegenerative diseases and elaborated that VGCCs can mediate the influx of toxic amounts of Ca^2+^ into neurons. This toxic Ca^2+^ influx depends on Ca^2+^ channel density and/or activity and increases during aging, chronic hypoxia, or exposure to Aβ peptides. Some data demonstrated a beneficial effect of drugs blocking VGCCs in various neurovascular and neurodegenerative diseases [225]. However, the differentiation between potentially “harmless” and “dangerous” VGCCs in neurodegenerative diseases and AD in particular turned out to be a sophisticated issue.

Several studies have suggested that soluble forms of Aβ facilitate influx through Ca^2+^-conducting ion channels into the plasma membrane, leading to excitotoxic neurodegeneration [226]. CCBs can attenuate Aβ-induced neuronal damage in vitro and turned out to be neuroprotective in animal models [226]. Interestingly, several CCBs have been evaluated in clinical trials of dementia, and the outcome was heterogeneous. Some DHPs, such as nimodipine or nilvadipine, prevented cognitive decline in various trials, whereas other CCBs failed to do so [206]. Importantly, in trials with a positive outcome, reduction of blood pressure did not seem to be effective in preventing dementia, suggesting an intrinsic protective impact on neurons. An optimization of CCBs for the treatment of dementia may involve increased selectivity for presynaptic VGCCs and improved affinity to the inactivated channel state [226]. Peters et al. (2014) carried out a systematic literature review on the use of CCBs and the relation to cognitive decline/dementia in older patients. The authors also concluded that there is no obvious evidence that administration of CCBs increases or decreases the susceptibility of cognitive decline or dementia in patients of older age and that robust clinical trials are necessary for the future to address this question [227]. Contemporaneously, Saravanaraman et al. (2014) reviewed the potential role of CCBs as cognitive enhancers and their use as drugs in the prevention or treatment of AD. CCBs were judged to genuinely exhibit cognitive-enhancing properties and diminish the risk of dementia, particularly in AD [228]. Lovell et al. (2015) illustrated that previous epidemiologic studies suggested a protective role of antihypertensive drugs against cognitive decline. The authors demonstrated that treatment with DHPs such as nifedipine led to a significant reduction of Aβ1–42 levels without an obvious decrease in cell viability [229]. In summary, these data suggest that the use of CCBs significantly diminishes the degree of progression to dementia and may minimize Aβ1–42 formation [229]. Again, these findings could be explained by a shift from the tonic mode to the burst mode upon DHP treatment leading to decreased tonic inhibition in the septohippocampal system, less reduction in GBRs, stabilization of APP/GBR complexes, and, consequently, diminished Aβ production.

Interestingly, support has recently emerged from clinical trials using T-type CCBs to treat AD, after some success with L-type CCBs in animal models of AD [230]. Furthermore, the assumption that antihypertensive drugs reduce the risk of dementia remains controversial. Recently, a database study of antihypertensives elaborated that while angiotensin-converting enzyme inhibitor and beta-blocker use was inversely associated with incident dementia, CCB use was positively associated with cognitive deficits [231]. The complex etiopathogenesis of AD and the multiple functional implications of VGCCs might have led to the misconception that cardiovascular manipulation is the only strategical target of CCBs relevant for AD. Instead, specific VGCC subgroups and entities differentially affect Aβ pathogenesis by influencing the amyloidogenic environment.

In the Cochrane review by Liu and Wang (2021), the authors assessed the efficacy and tolerability of pharmacological interventions via antiepileptic drugs (AEDs) for the treatment of epilepsy in people with AD. The Cochrane Register of Studies (CRS Web) and MEDLINE (Ovid, 1946 to 31 July 2020) include randomized or quasi-randomized controlled trials (RCTs) from PubMed, EMBASE, ClinicalTrials.gov, the World Health Organization International Clinical Trials Registry Platform (ICTRP), the Cochrane Central Register of Controlled Trials (CENTRAL), and the Specialized Registers of Cochrane Review Groups, including Cochrane Epilepsy. Further published, unpublished, and ongoing trials were identified by contacting trial authors and pharmaceutical companies [232]. It was found that levetiracetam could lead to improvement in cognition and lamotrigine to a relief in depression. On the other hand, phenobarbital and lamotrigine could aggravate cognition, and levetiracetam and phenobarbital could deteriorate mood [232]. The risk of bias due to allocation, blinding, and selective reporting was indistinct. The authors concluded that there is currently insufficient evidence to support levetiracetam, phenobarbital, or lamotrigine for the pharmacological treatment of epilepsy in patients suffering from AD. No significant differences were found between levetiracetam, phenobarbital, and lamotrigine in efficacy and tolerability in the specific therapeutic settings [232].

Seibert et al. (2021) carried out a literature search in MEDLINE, Embase, and CENTRAL for RCTs of drug therapy of AD in patients with severe functional impairments to evaluate the efficacy and safety of pharmacotherapy. From this study, there is strong evidence for increased risk of drug-induced adverse reactions in these patients [233]. Regarding anticonvulsants, four studies were included for evaluation of safety and efficacy for aggressive or agitated behavior in patients with AD, vascular, or mixed dementia. Three of the RCTs were related to carbamazepine treatment [234,235,236], and the fourth one assessed the implications of valproate administration [237]. Seibert et al. (2021) also concluded that there is still a lack of evidence, which makes it hard to provide valid recommendations for drug therapy of AD with behavioral and psychological symptoms. There is still a strong need for additional clinical trials in the future [233]. Apart from clinical trials, the question of neuroprotective or pro-neurodegenerative effects of CCBs can be evaluated via in vitro and in vivo models. These models include, i.e., the ischemia model (via the oxygen-glucose deprivation), neuronal cell line models, peripheral neuropathy models, and hearing loss models.

Given these findings, special attention needs to be paid to the application of T-type CCBs in the elderly. In general, T-type CCBs could be beneficial in various CNS-related diseases, such as neuropathic pain, epilepsy (e.g., absence epilepsy), PD, sleep disorder (sleep cycle dysregulation, insomnia), autism, and essential tremor, as well as cancer/tumor cycle regulation [93,94,103,238,239,240,241,242].

Many blockers with predominant T-type antagonism are indeed used for the treatment of various human diseases, and, in many instances, the T-type blocking properties constitute only one aspect of their pharmacodynamic profile. Importantly, only with one exception [243], the neuroprotective effect of CCBs is not solely related to direct blockage of T-type mediated Ca^2+^ currents. On the contrary, for most T-type CCBs, T-type inhibition represents only one pharmacodynamic aspect, whereas multiple other ion channels and/or signaling cascades are also targeted [119]. Thus, it must be noted that the classification of drugs as T-type Ca^2+^ channel blockers is critical and potentially misleading. Clearly, it is mandatory to differentiate the putative neuroprotective effects of some so-called T-type CCBs from the inhibitory effects on T-type VGCCs, based on multiple additional competitive/overlapping mechanisms. The latter has been summarized, for example, in detail for zonisamide [119].

Antiepileptic drugs represent a group of pharmaceuticals that often affect T-type VGCCs. However, it must be emphasized that most AEDs exert polypharmacodynamic properties, i.e., they have a multitarget character, and drug-dependently affect, e.g., voltage-gated sodium channels, potassium channels, glutamate receptors, etc. Here, we selectively focus on the T-type Ca^2+^ channel blocking properties. Beghi and Beghi (2020) provided a comprehensive overview to illustrate the frequency and trends of the comorbidity of epilepsy and dementia and the effects of AEDs on cognitive functions [244]. Importantly, Taipale et al. (2018) evaluated the association between regular AED use and incident dementia based on a case–control analysis from a Finnish public health register and German health insurance data. This analysis included individuals with dementia of any type (German data, N = 20,325) and AD (Finnish data, N = 70,718) [245]. The analysis of the association between AED use and dementia elicited that AED administration was more frequent in individuals with dementia compared to controls. The latter is in line with previous observations of increased incidence of epilepsy in AD patients (see above). Strikingly, the regular use of AEDs was accompanied by a significantly greater risk of incident dementia compared to no AED use. Additionally, the increased risk of dementia upon AED use turned out to be dose-dependent and was more prominent in those AEDs that exhibit cognitive adverse effects [245]. Taipale et al. (2018) speculated that effects on GABAergic neurotransmission might account for these phenomena. Given the new findings on the functional interdependence between T-type VGCCs, the GABAergic system, APP/GBR complexes, and Aβ formation outlined in this review, it seems likely that pharmacological blockage of LVA T-type VGCCs is a critical factor in the proamyloidogenic scenario associated with increased risk of developing AD, although this needs to be clearly proven in the future. Whereas blockage of LVA T-Type Ca^2+^ channels apparently increases the risk of developing AD and aggravates its progression based on the model developed here, augmentation of LVA T-type Ca^2+^ currents might successfully counteract this devastating process. Notably, studies from cancer research that identified the cacna1g gene encoding Ca_v_3.1 as a tumor suppressor gene point in this direction. It has been shown that age-related cacna1g promoter hypermethylation causes a decrease in Ca_v_3.1 expression in a number of peripheral cancers [246,247]. Interestingly, the new anticancer compound ST101 (a small peptide antagonist of C/EBPβ) was reported to inhibit Aβ generation and enhance cognition processes in 3xTg-AD mice. Strikingly, it turned out that ST101 acts via enhancement of T-type VGCCs [121,248]. In 3xTg AD mice carrying APPKM670/671NL, PS1M146V, and TAUP301L mutations, 2-month administration of ST101 was reported to diminish Aβ accumulation and enhance spatial memory [248]. Interestingly, ST101 triggered APP processing at a new cleavage site, which resulted in a novel C-terminal 17 kDa fragment [248]. A phase II clinical trial in the US suggested that ST101 should be effective and safe in combination treatment of AD [249]. A spiroimidazopyridine derivative of ST101, i.e., SAK3, turned out to be an even more potent enhancer of T-type Ca^2+^ current [250]. Chronic three months treatment of 8- and 12-month-old APP23 (APP KM670/671NL) mice using SAK3 resulted in reduced soluble and insoluble Aβ levels and Aβ deposits. The novel therapeutic candidate SAK3 was reported to stimulate neuronal Ca_v_3.1 and Ca_v_3.3 VGCCs [122]. In addition, SAK3 was shown to inhibit both accumulation and aggregation of Aβ in APP mutant mice [251]. SAK3 was speculated to serve as a novel disease-modifying drug candidate in AD therapy. Fukunaga et al. (2019) proposed a potential mechanism of how T-type Ca^2+^ channel stimulation could prevent Aβ deposit formation [250]. Given the functional expression of T-type VGCCs in GABAergic neurons, inhibition of these channels can impair ACh- or dopamine-evoked GABA release in the cortex and hippocampus [100,250,252,253]. Importantly, this phenomenon would further aggravate the functional disinhibition in the septohippocampal system, as described in our model (see also Figure 1). On the other hand, stimulation of T-Type VGCCs, e.g., via ST101 or SAK3, enhanced ACh-mediated GABA release in the rat hippocampus [254]. Furthermore, T-type VGCC activation can increase ACh release from cholinergic neurons and trigger enhanced cholinergic and/or glutamatergic transmission on postsynaptic cells. The latter can lead to stimulation of CaMKII and enhance proteasome activation. Notably, soluble Aβ/Aβ oligomers can significantly impair the ubiquitin-proteasome system (UPS) in the AD brain and diminish prompt degradation of Aβ oligomers and aggregated tau [255,256,257,258]. UPS was further shown to serve as a potential target in AD treatment [259,260,261]. Importantly, CaMKII activation can stimulate proteasomes and eliminate misfolded proteins/aggregates in neurodegenerative diseases. Clearly, additional studies are necessary to further elaborate the detailed mechanisms of CaMKII-mediated proteasome stimulation and subsequent proteolytic degradation of Aβ oligomers or larger aggregates. It should be noted that administration of NNC 55-0396, a structural analog of mibefradil, which serves as a T-type VGCC antagonist (Ca_v_3.1: IC_50_ 7 μmol/L [262]), resulted in increased CNS levels of Aβ1–40/42 in 3xTg Alzheimer mice, accordingly [201].

Besides the direct potential role of Ca_v_3.x VGCCS in APP/GBR stabilization and CaMKII/proteasome-mediated Aβ degradation, T-type Ca^2+^ channel activation was reported to be involved in hippocampal neurogenesis as well. In AD patients, behavioral and psychological symptoms of dementia (BPSD) were speculated to result from impaired neurogenesis, e.g., in the dentate gyrus [263,264,265]. In mice, administration of mecamylamine, which serves as a nonselective nicotinic ACh receptor antagonist, was shown to inhibit ST101-mediated neurogenesis in the dentate gyrus [264,266]. The T-type VGCC stimulator SAK3 was further demonstrated to trigger proliferation of hippocampal dentate gyrus cells and support their survival [267]. With ST101 and SAK3 serving as potential therapeutic candidates for future AD treatment, the modulation, i.e., enhancement, of Ca_v_3.x T-type Ca^2+^ current has to move into a broad focus in drug research and development in AD.

It is important to note that, given the pathophysiological implications of T-type Ca^2+^ channels in many neuropsychiatric disorders, strong efforts are being made to establish novel T-type CCBs. Mibefradil was the first compound marketed for selective blockage of T-type VGCCs but was withdrawn soon after initial licensing. Since that time, substantial efforts have been made to identify and characterize selective T-type VGCC blockers [268,269,270]. Nam (2018) reviewed 43 patents describing organic small molecules as T-type Ca^2+^ channel blockers published since 2012 [268]. A similar patent review was published in 2011 [271]. Recent patents include (i) fused bicyclic pyridine/pyrimidine derivatives, (ii) phenylpyrimidinone and phenyltetrahydropyridine derivatives, (iii) triazinone derivatives, (iv) phenylflavone/tetrahydronaphthalene derivatives, (v) carbazole/piperidine/piperazine-bearing derivatives, (vi) arylsulfonamide derivatives, and (vii) heteroaromatic amides. Triazinone derivatives, carbazole compounds, and aryl triazole/imidazole amide derivatives were identified as potential blockers of Ca_v_3.1 and Ca_v_3.2 T-type VGCCs [268]. There are ongoing clinical trials for some of these promising candidates. However, given the model presented here, only T-type Ca^2+^ channel enhancers/activators harbor the capability to stabilize APP/GBR receptors (and promote Aβ degradation via CaMKII-mediated proteasome activation) and generate an antiamyloidogenic environment. Thus, it is mandatory to put a specific focus also on T-type Ca^2+^ channel enhancers in drug research and development in the future.

## 10. Conclusions

Ablation of Ca_v_3 T-type VGCCs such as Ca_v_3.1 and Ca_v_3.2 has been related to altered atropine-sensitive type II theta activity and modified theta architecture in the CA1 region of mice. In this review, we bridged the gap between VGCCs and the GABAergic system by demonstrating that tonic inhibition of hippocampal GABAergic interneurons and subsequent disinhibition of pyramidal cells account for theta alterations and cause compensatory changes in the GABAergic receptor and transmission system.

Based on the preclinical and clinical findings depicted above, we propose a specific model of functional disinhibition within the septohippocampal GABAergic pathway that is responsible for the observed increase in atropine-sensitive type II theta upon ablation of LVA Ca_v_3 Ca^2+^ channels. These alterations could serve as a blueprint in other brain regions as well and have tremendous proamyloidogenic implications for the entire CNS. The detailed sequential steps of our model in causal assumption are as follows (see also Figure 2 and Figure 3):

(i) Ca_v_3.2 VGCCs are dominantly expressed (>Ca_v_3.1 VGCCs) in the septum and hippocampus.
(ii) Global ablation and septum-specific inactivation of Ca_v_3.1 results in a functional shift from the (rebound) burst firing mode to the tonic mode in GABAergic interneurons.
(iii) As LVA Ca_v_3 T-type Ca^2+^ channels generate the low-threshold Ca^2+^ current (LTCC) underlying LTCS, ablation of Ca_v_3.1 and Ca_v_3.2 is hypothesized to dramatically impair the (rebound) burst firing mode (facultative neuronal pacemaker activity) in septal GABAergic interneurons as well.
(iv) Consequently, ablation of Ca_v_3.2 and/or Ca_v_3.1 in septal inhibitory GABAergic neurons favors the tonic firing mode and tonic inhibition of hippocampal interneurons.
(v) It is hypothesized that the tonic inhibition of hippocampal GABAergic interneurons (e.g., Chandelier cells, Basket cells) mediates functional disinhibition in the septohippocampal system with enhanced activation pattern in pyramidal neurons and augmented theta activity.
(vi) This phenomenon exhibits clear sensitivity towards muscarinic receptor modulation (e.g., via urethane) and suggests that atropine-sensitive type II theta is likely to be affected.
(vii) Ca_v_3.2 VGCC ablation causes a reduction of GABA receptor subunit transcript levels and presumably affects expression/protein levels as well.
(viii) As GBRs were shown to form microcomplexes with APP and be involved in APP stabilization, Ca_v_3.2 ablation potentially destabilizes GBR1a/APP microassemblies.
(ix) This destabilization is supposed to increase Aβ levels and generate a proamyloidogenic effect/proamyloidogenic environment.
(x) In vivo and in vitro long-term studies in Ca_v_3.2-deficient mice might reveal the potential role of Ca_v_3.2 VGCCs in a proamyloidogenic environment composed of LVA T-type Ca^2+^ channels, GABA A and B receptors and APP in the septohippocampal system.
(xi) Aβ, as a cleavage product of APP, was shown to exert differential effects on VGCCs. Given the hypothesis presented above it might be speculated that destabilization of GBR/APP complexes increases APP cleavage, Aβ generation and T-type VGCC inhibition. The latter might further aggravate neuronal degradation in a type of vicious cycle.

It needs to be emphasized that the model proposed above has not yet been proven by the use of Ca_v_3.x T-type CCBs described in this review. It is also important to consider that dysregulation of Ca_v_3 VGCCs in this setting could be related to alterations, e.g., in voltage-gated Na^+^ channels or other Ca^2+^ channel entities. However, the model outlined above might turn out to be clinically relevant. Notably, the functional age-related interdependence between Aβ and LVA Ca_v_3.x T-type Ca^2+^ channels is critical on three different levels: (i) pharmaceutical agents with Ca_v_3.x T-type blocking properties are used worldwide. The fact that Ca_v_3 channels exhibit an age-related reduction in expression, particularly in the brain, can have a substantial influence on the effectiveness in the elderly and might be a reason why such drugs are less effective in older age. (ii) Numerous drugs that have been approved by (supra)national competent authorities exhibit T-type blocking properties. This includes drugs, e.g., in the cardiovascular field, as well as in neuropsychiatry. In the latter, a specific focus is on AEDs, most of which exert multitarget properties. Importantly, some of them, such as suximides (e.g., ethosuximide, methsuximide, and its active metabolite α-methyl-α-phenylsuccinimide), zonisamide, trimethadione, and valproate, have Ca_v_3.x T-type blocking characteristics [272,273] (see also Table 2). There are pharmacoepidemiological signals suggesting that drugs with a T-type blocking pharmacodynamic profile could increase the risk of developing dementia. Studies of others and from our own group propose that reduction in T-type Ca^2+^ current affects GABAergic transmission with decreased GBR expression, tonic disinhibition, and APP/GBR destabilization (Figure 3A). The model presented here could thus explain why administration of drugs with T-type blocking properties could be associated with an increased risk of dementia. Although T-type blockade was judged to be potentially beneficial in τ pathogenesis, it is generally accepted that Aβ accumulation precedes τ hyperphosphorylation. Therefore, T-type Ca^2+^ channel blockade is likely to exert a net proamyloidogenic environment in older adults [201]. (iii) Conversely, agonistic pharmacological interference with Ca_v_3.x T-type Ca^2+^ channels in the aged brain may provide innovative therapeutic approaches for the prevention of AD. This strategy is further strengthened by the T-type Ca^2+^-channel-mediated stimulation of CaMKII, proteasome activation, and, subsequently, enhanced Aβ degradation (see also Figure 3B). Based on our model proposed here, Ca_v_3.1 and Ca_v_3.2 VGCC downregulation could trigger the accumulation of Aβ in a proamyloidogenic environment. Thus, LVA Ca_v_3.x T-type agonists could exert preventive therapeutic action in AD and serve as novel targets in drug research and development in the future.

**Table 2:** Effects of antiepileptic drugs, general anesthetics, and antipsychotics on Ca_v_3.1, Ca_v_3.2, and Ca_v_3.3 VGCCs. This table lists a selection of AEDs, anesthetics, and antipsychotics that were reported to exert antagonistic effects on Ca_v_3.x T-type VGCCs. In addition, IC_50_ values are listed for the individual drugs, together with drug-related therapeutic plasma concentrations. Some experimental and licensed CCBs, e.g., mibefradil, amlodipine, nimodipine, isradipine, nifedipine, and verapamil, exhibit IC_50_ values for Ca_v_3.1–3.3 in the micromolar range, which is above the therapeutic plasma concentrations. Note that blockage of Ca_v_3.x VGCCs is known to be state-dependent for many drugs (e.g., for ethosuximides or MPS), with higher affinity for the inactive state. Thus, the membrane potential (or holding potential) has a tremendous impact on steady-state inactivation and IC_50_ values, which needs to be considered when interpreting IC_50_ values and therapeutic plasma concentrations. Clearly, most of the drugs listed here, particularly AEDs, have a multitarget character and modulate other voltage-gated ion channels or ligand-gated ionotropic and metabotropic ion channels as well [274]. For references, see [105,115,119,272,273,275,276,277,278,279,280,281,282,283,284,285].

	Ca_v_3.1	Ca_v_3.2	Ca_v_3.3	Ca_v_3.x	Therapeutic Plasma Concentration
**AED**					
**Phenytoin**	IC_50_ = 74 μmol/L	-----	-----	-----	80 μmol/L
**Ethosuximide**	IC_50_ > 3 mmol/L	IC_50_ < 300 μmol/L	-----	IC_50_ = 0.3–1 mmol/L	700 μmol/L
**MPS**	IC_50_ = 1.95 mmol/L	IC_50_ = 3.03 mmol/L	IC_50_ = 1.82 mmol/L	-----	700 μmol/L
**Zonisamide**	14% block at 50 mmol/L	17% block at 100 μmol/L	10% block at 50 μmol/L	IC_50_ = 0.05–0.5 mmol/L	50–100 mmol/L
**Lamotrigine**	10% block at 100 μmol/L	-----	no effect	-----	40 μmol/L
**Sipatrigine**	IC_50_ ≈ 15 μmol/L	IC_50_ ≈ 15 μmol/L	IC_50_ = 14 μmol/L	-----	-----
**Valproate**	max. block 10% at 1 mmol/L	-----	-----	-----	300–600 μmol/L
**Anaesthetics**					
**Propofol**	IC_50_ = 21 μmol/L	-----	-----	-----	50 μmol/L
**Etomidate**	IC_50_ = 161 μmol/L	-----	-----	-----	2 μmol/L
**Isoflurane**	IC_50_ = 277 μmol/L	-----	-----	-----	100 μmol/L
**Ketamine**	IC_50_ = 1.2 mmol/L	-----	-----	-----	20 μmol/L
**Thiopental**	IC_50_ = 280 μmol/L	-----	-----	-----	20 μmol/L
**Pentobarbital**	IC_50_ = 310 μmol/L	-----	-----	-----	22 μmol/L
**Phenobarbital**	IC_50_ = 1.5 mmol/L	-----	-----	-----	170 μmol/L
**Antipsychotics**					
**Pimozide**	IC_50_ = 35 nmol/L IC_50_ = 2 μmol/L max. block 40%	IC_50_ = 54 nmol/L IC_50_ = 15 μmol/L max. block 30%	IC_50_ = 30 nmol/L IC_50_ = 1.6 μmol/L max. block 25%	-----	40 nmol/L
**Haloperidol**	IC_50_ ≈ 1 μmol/L IC_50_ = 1.5 μmol/L max. block 60%	IC_50_ ≈ 1 μmol/L IC_50_ = 3 μmol/L max. block 55%	IC_50_ ≈ 1 μmol/L IC_50_ = 35 μmol/L max. block 86%	-----	0.5 μmol/L
**Fluspirilene**	IC_50_ = 12 μmol/L max. block 80%	IC_50_ = 7 μmol/L max. block 80%	IC_50_ = 12 μmol/L max. block 62%	-----	-----
**Flunarizine**	IC_50_ ≤ 1 μmol/L	IC_50_ > 1 μmol/L	IC_50_ ≤ 1 μmol/L	-----	0.25 μmol/L
**Penfluoridol**	IC_50_ = 93 nmol/L	IC_50_ = 64 nmol/L	IC_50_ = 72 nmol/L	-----	40 nmol/L

Clearly, additional studies are mandatory to unravel the potential functional interdependence between T-type VGCCs, the GABAergic system, and APP and their role in the etiopathogenesis of AD. Furthermore, one should be aware that other voltage-gated ion channels and transmitter systems, e.g., the cholinergic and glutamatergic system, interfere with the model presented here. However, for reasons of lucidity, this interaction was not further elaborated here. From a future pharmacovigilance perspective, it is essential to further analyze and monitor the potential pharmacoepidemiological and mechanistic link between LVA Ca_v_3.x T-type VGCCs, the GABAergic system, and APP/Aβ that might be involved in generating a proamyloidogenic environment in the brain. Thus, the application of Ca_v_3.x T-type channel blockers should be critically monitored and evaluated for their potential proamyloidogenic action, particularly when used in older patients or those already suffering from mild cognitive impairment (MCI) or AD.

## Data Availability

Not applicable.

## Abbreviations

Aβ	amyloid-beta
AChR	acetylcholine receptor
AD	Alzheimer’s disease
AED	antiepileptic drug
AHP	afterhyperpolarization
AMPA	α-amino-3-hydroxy-5-methyl-4-isoxazolepropionic acid
APP	amyloid precursor protein
ASD	autism spectrum disorder
BACE	β-site APP cleaving enzyme
BK	big conductance K^+^ channels
CA	cornu ammonis
CENTRAL	Cochrane Central Register of Controlled Trials
CCB	calcium channel blocker
CNS	central nervous system
CRS	Cochrane Register of Studies
DAG	diacylglycerol
Dynlt1b	dynein light chain Tctex-type 1
DHP	dihydropyridine
EC	entorhinal cortex
ER	endoplasmic reticulum
Gαq/11	α-subunit of the heterotrimeric G_q/11_ protein
GABA	gamma-aminobutyric acid
GBR	GABA B receptor
GPCR	G-protein-coupled receptor
HC	hippocampus
HVA	high voltage-activated
ICTRP	International Clinical Trials Registry Platform
InsP3	inositoltrisphosphate
JIP	c-Jun N-terminal kinase-interacting protein
LTCC	low-threshold Ca^2+^ current
LTCS	low-threshold Ca^2+^ spike
LTP	long-term potentiation
LVA	low voltage-activated
mAChR	muscarinic acetylcholine receptor
MCI	mild cognitive impairment
M1/M3	muscarinic receptor type 1/3
MS	medial septum
MS-DBB	medial septum-diagonal band of Broca
NgR	Nogo receptor family
NMDA	N-methyl-D-aspartate
NPY	neuropeptide Y
OLM	outer lacunosum-moleculare layer
PCG	posterior cingulate gyrus
PD	Parkinson’s disease
PKC	protein kinase C
PLC	phospholipase C
PV	parvalbumin
qPCR	quantitative polymerase chain reaction
RCT	randomized controlled trial
RyR	ryanodine receptor
REM	rapid eye movement
ROS	reactive oxygen species
R-type	“resistant”-type Ca^2+^ channel
sAPP	secreted APP
SST	somatostatin
SFG	superior frontal gyrus
T-type	“transient”-type Ca^2+^ channel
VGCC	voltage-gated Ca^2+^ channel

## References

[1] Vanderwolf C.H. (1969). Hippocampal electrical activity and voluntary movement in the rat. Electroencephalogr. Clin. Neurophysiol..

[2] Davies P., Maloney A.J. (1976). Selective loss of central cholinergic neurons in Alzheimer’s disease. Lancet.

[3] O’Keefe J., Nadel L. (1978). The Hippocampus as a Cognotive Map.

[4] Whitehouse P.J., Price D.L., Struble R.G., Clark A.W., Coyle J.T., Delon M.R. (1982). Alzheimer’s disease and senile dementia: Loss of neurons in the basal forebrain. Science.

[5] Kahana M.J., Seelig D., Madsen J.R. (2001). Theta returns. Curr. Opin. Neurobiol..

[6] Vertes R.P., Hoover W.B., Viana Di Prisco G. (2004). Theta rhythm of the hippocampus: Subcortical control and functional significance. Behav. Cogn. Neurosci. Rev..

[7] Vertes R.P. (2005). Hippocampal theta rhythm: A tag for short-term memory. Hippocampus.

[8] MacDonald C.J., Carrow S., Place R., Eichenbaum H. (2013). Distinct hippocampal time cell sequences represent odor memories in immobilized rats. J. Neurosci..

[9] Jacobs J. (2014). Hippocampal theta oscillations are slower in humans than in rodents: Implications for models of spatial navigation and memory. Philos. Trans. R. Soc. Lond. B Biol. Sci..

[10] Jacobs J., Miller J., Lee S.A., Coffey T., Watrous A.J., Sperling M.R., Sharan A., Worrell G., Berry B., Lega B. (2016). Direct Electrical Stimulation of the Human Entorhinal Region and Hippocampus Impairs Memory. Neuron.

[11] Kraus B.J., Brandon M.P., Robinson R.J., Connerney M.A., Hasselmo M.E., Eichenbaum H. (2015). During Running in Place, Grid Cells Integrate Elapsed Time and Distance Run. Neuron.

[12] Burwell R.D., Amaral D.G. (1998). Cortical afferents of the perirhinal, postrhinal, and entorhinal cortices of the rat. J. Comp. Neurol..

[13] Gu Z., Yakel J.L. (2011). Timing-dependent septal cholinergic induction of dynamic hippocampal synaptic plasticity. Neuron.

[14] Remondes M., Schuman E.M. (2004). Role for a cortical input to hippocampal area CA1 in the consolidation of a long-term memory. Nature.

[15] Canto C.B., Wouterlood F.G., Witter M.P. (2008). What does the anatomical organization of the entorhinal cortex tell us?. Neural Plast..

[16] Pelkey K.A., Chittajallu R., Craig M.T., Tricoire L., Wester J.C., McBain C.J. (2017). Hippocampal GABAergic Inhibitory Interneurons. Physiol. Rev..

[17] Buzsaki G. (2002). Theta oscillations in the hippocampus. Neuron.

[18] Buzsaki G., Buhl D.L., Harris K.D., Csicsvari J., Czeh B., Morozov A. (2003). Hippocampal network patterns of activity in the mouse. Neuroscience.

[19] Oddie S.D., Bland B.H. (1998). Hippocampal formation theta activity and movement selection. Neurosci. Biobehav. Rev..

[20] Borhegyi Z., Varga V., Szilagyi N., Fabo D., Freund T.F. (2004). Phase segregation of medial septal GABAergic neurons during hippocampal theta activity. J. Neurosci..

[21] Simon A.P., Poindessous-Jazat F., Dutar P., Epelbaum J., Bassant M.H. (2006). Firing properties of anatomically identified neurons in the medial septum of anesthetized and unanesthetized restrained rats. J. Neurosci..

[22] Varga V., Hangya B., Kranitz K., Ludanyi A., Zemankovics R., Katona I., Shigemoto R., Freund T.F., Borhegyi Z. (2008). The presence of pacemaker HCN channels identifies theta rhythmic GABAergic neurons in the medial septum. J. Physiol..

[23] Lubenov E.V., Siapas A.G. (2009). Hippocampal theta oscillations are travelling waves. Nature.

[24] Ma S., Olucha-Bordonau F.E., Hossain M.A., Lin F., Kuei C., Liu C., Wade J.D., Sutton S.W., Nunez A., Gundlach A.L. (2009). Modulation of hippocampal theta oscillations and spatial memory by relaxin-3 neurons of the nucleus incertus. Learn. Mem..

[25] Takano Y., Hanada Y. (2009). The driving system for hippocampal theta in the brainstem: An examination by single neuron recording in urethane-anesthetized rats. Neurosci. Lett..

[26] Hangya B., Borhegyi Z., Szilagyi N., Freund T.F., Varga V. (2009). GABAergic neurons of the medial septum lead the hippocampal network during theta activity. J. Neurosci..

[27] Goutagny R., Manseau F., Jackson J., Danik M., Williams S. (2008). In vitro activation of the medial septum-diagonal band complex generates atropine-sensitive and atropine-resistant hippocampal theta rhythm: An investigation using a complete septohippocampal preparation. Hippocampus.

[28] Muller R., Struck H., Ho M.S., Brockhaus-Dumke A., Klosterkotter J., Broich K., Hescheler J., Schneider T., Weiergraber M. (2012). Atropine-sensitive hippocampal theta oscillations are mediated by Ca_v_2.3 R-type Ca^2+^ channels. Neuroscience.

[29] Gillies M.J., Traub R.D., LeBeau F.E., Davies C.H., Gloveli T., Buhl E.H., Whittington M.A. (2002). A model of atropine-resistant theta oscillations in rat hippocampal area CA1. J. Physiol..

[30] Buzsaki G., Czopf J., Kondakor I., Kellenyi L. (1986). Laminar distribution of hippocampal rhythmic slow activity (RSA) in the behaving rat: Current-source density analysis, effects of urethane and atropine. Brain Res..

[31] Chuang S.C., Bianchi R., Kim D., Shin H.S., Wong R.K. (2001). Group I metabotropic glutamate receptors elicit epileptiform discharges in the hippocampus through PLCbeta1 signaling. J. Neurosci..

[32] Kramis R., Vanderwolf C.H., Bland B.H. (1975). Two types of hippocampal rhythmical slow activity in both the rabbit and the rat: Relations to behavior and effects of atropine, diethyl ether, urethane, and pentobarbital. Exp. Neurol..

[33] Vanderwolf C.H. (1988). Cerebral activity and behavior: Control by central cholinergic and serotonergic systems. Int. Rev. Neurobiol..

[34] Shin J., Gireesh G., Kim S.W., Kim D.S., Lee S., Kim Y.S., Watanabe M., Shin H.S. (2009). Phospholipase C beta 4 in the medial septum controls cholinergic theta oscillations and anxiety behaviors. J. Neurosci..

[35] Madison D.V., Lancaster B., Nicoll R.A. (1987). Voltage clamp analysis of cholinergic action in the hippocampus. J. Neurosci..

[36] Brown D.A. (2010). Muscarinic acetylcholine receptors (mAChRs) in the nervous system: Some functions and mechanisms. J. Mol. Neurosci..

[37] Colino A., Halliwell J.V. (1993). Carbachol potentiates Q current and activates a calcium-dependent non-specific conductance in rat hippocampus in vitro. Eur. J. Neurosci..

[38] Shin J., Kim D., Bianchi R., Wong R.K., Shin H.S. (2005). Genetic dissection of theta rhythm heterogeneity in mice. Proc. Natl. Acad. Sci. USA.

[39] Magee J.C., Johnston D. (1995). Synaptic activation of voltage-gated channels in the dendrites of hippocampal pyramidal neurons. Science.

[40] Magee J.C., Carruth M. (1999). Dendritic voltage-gated ion channels regulate the action potential firing mode of hippocampal CA1 pyramidal neurons. J. Neurophysiol..

[41] Yasuda R., Sabatini B.L., Svoboda K. (2003). Plasticity of calcium channels in dendritic spines. Nat. Neurosci..

[42] Giessel A.J., Sabatini B.L. (2010). M1 muscarinic receptors boost synaptic potentials and calcium influx in dendritic spines by inhibiting postsynaptic SK channels. Neuron.

[43] Bloodgood B.L., Sabatini B.L. (2007). Nonlinear regulation of unitary synaptic signals by Ca_v_2.3 voltage-sensitive calcium channels located in dendritic spines. Neuron.

[44] Catterall W.A., Leal K., Nanou E. (2013). Calcium channels and short-term synaptic plasticity. J. Biol. Chem..

[45] Catterall W.A. (2011). Voltage-gated calcium channels. Cold Spring Harb. Perspect. Biol..

[46] Nanou E., Catterall W.A. (2018). Calcium Channels, Synaptic Plasticity, and Neuropsychiatric Disease. Neuron.

[47] Weiergraber M., Kamp M.A., Radhakrishnan K., Hescheler J., Schneider T. (2006). The Ca_v_2.3 voltage-gated calcium channel in epileptogenesis—Shedding new light on an enigmatic channel. Neurosci. Biobehav. Rev..

[48] Weiergraber M., Henry M., Krieger A., Kamp M., Radhakrishnan K., Hescheler J., Schneider T. (2006). Altered seizure susceptibility in mice lacking the Ca_v_2.3 E-type Ca^2+^ channel. Epilepsia.

[49] Weiergraber M., Henry M., Radhakrishnan K., Hescheler J., Schneider T. (2007). Hippocampal seizure resistance and reduced neuronal excitotoxicity in mice lacking the Ca_v_2.3 E/R-type voltage-gated calcium channel. J. Neurophysiol..

[50] Wilson S.M., Toth P.T., Oh S.B., Gillard S.E., Volsen S., Ren D., Philipson L.H., Lee E.C., Fletcher C.F., Tessarollo L. (2000). The status of voltage-dependent calcium channels in alpha 1E knock-out mice. J. Neurosci..

[51] Sochivko D., Pereverzev A., Smyth N., Gissel C., Schneider T., Beck H. (2002). The Ca_v_2.3 Ca^2+^ channel subunit contributes to R-type Ca^2+^ currents in murine hippocampal and neocortical neurones. J. Physiol..

[52] Sochivko D., Chen J., Becker A., Beck H. (2003). Blocker-resistant Ca^2+^ currents in rat CA1 hippocampal pyramidal neurons. Neuroscience.

[53] Vilaro M.T., Mengod G., Palacios G., Palacios J.M. (1993). Receptor distribution in the human and animal hippocampus: Focus on muscarinic acetylcholine receptors. Hippocampus.

[54] Vilaro M.T., Mengod G., Palacios J.M. (1993). Advances and limitations of the molecular neuroanatomy of cholinergic receptors: The example of multiple muscarinic receptors. Prog. Brain Res..

[55] Levey A.I., Edmunds S.M., Koliatsos V., Wiley R.G., Heilman C.J. (1995). Expression of m1-m4 muscarinic acetylcholine receptor proteins in rat hippocampus and regulation by cholinergic innervation. J. Neurosci..

[56] Rouse S.T., Marino M.J., Potter L.T., Conn P.J., Levey A.I. (1999). Muscarinic receptor subtypes involved in hippocampal circuits. Life Sci..

[57] Bannister R.A., Melliti K., Adams B.A. (2004). Differential modulation of Ca_v_2.3 Ca^2+^ channels by Galphaq/11-coupled muscarinic receptors. Mol. Pharmacol..

[58] Kamatchi G.L., Franke R., Lynch C., Sando J.J. (2004). Identification of sites responsible for potentiation of type 2.3 calcium currents by acetyl-beta-methylcholine. J. Biol. Chem..

[59] Tai C., Kuzmiski J.B., MacVicar B.A. (2006). Muscarinic enhancement of R-type calcium currents in hippocampal CA1 pyramidal neurons. J. Neurosci..

[60] Meza U., Bannister R., Melliti K., Adams B. (1999). Biphasic, opposing modulation of cloned neuronal alpha1E Ca channels by distinct signaling pathways coupled to M2 muscarinic acetylcholine receptors. J. Neurosci..

[61] Klockner U., Pereverzev A., Leroy J., Krieger A., Vajna R., Pfitzer G., Hescheler J., Malecot C.O., Schneider T. (2004). The cytosolic II-III loop of Ca_v_2.3 provides an essential determinant for the phorbol ester-mediated stimulation of E-type Ca^2+^ channel activity. Eur. J. Neurosci..

[62] Kuzmiski J.B., Barr W., Zamponi G.W., MacVicar B.A. (2005). Topiramate inhibits the initiation of plateau potentials in CA1 neurons by depressing R-type calcium channels. Epilepsia.

[63] Pemberton K.E., Hill-Eubanks L.J., Jones S.V. (2000). Modulation of low-threshold T-type calcium channels by the five muscarinic receptor subtypes in NIH 3T3 cells. Pflugers Arch..

[64] Zhang Y., Jiang X., Snutch T.P., Tao J. (2013). Modulation of low-voltage-activated T-type Ca^2+^ channels. Biochim. Biophys. Acta.

[65] Hildebrand M.E., David L.S., Hamid J., Mulatz K., Garcia E., Zamponi G.W., Snutch T.P. (2007). Selective inhibition of Ca_v_3.3 T-type calcium channels by Galphaq/11-coupled muscarinic acetylcholine receptors. J. Biol. Chem..

[66] Cribbs L.L., Gomora J.C., Daud A.N., Lee J.H., Perez-Reyes E. (2000). Molecular cloning and functional expression of Ca_v_3.1c, a T-type calcium channel from human brain. FEBS Lett..

[67] Cribbs L.L., Lee J.H., Yang J., Satin J., Zhang Y., Daud A., Barclay J., Williamson M.P., Fox M., Rees M. (1998). Cloning and characterization of alpha1H from human heart, a member of the T-type Ca^2+^ channel gene family. Circ. Res..

[68] Lee J.H., Gomora J.C., Cribbs L.L., Perez-Reyes E. (1999). Nickel block of three cloned T-type calcium channels: Low concentrations selectively block alpha1H. Biophys. J..

[69] Catterall W.A., Lenaeus M.J., Gamal El-Din T.M. (2020). Structure and Pharmacology of Voltage-Gated Sodium and Calcium Channels. Annu. Rev. Pharmacol. Toxicol..

[70] Zhuang H., Bhattacharjee A., Hu F., Zhang M., Goswami T., Wang L., Wu S., Berggren P.O., Li M. (2000). Cloning of a T-type Ca^2+^ channel isoform in insulin-secreting cells. Diabetes.

[71] Gomora J.C., Murbartian J., Arias J.M., Lee J.H., Perez-Reyes E. (2002). Cloning and expression of the human T-type channel Ca_v_3.3: Insights into prepulse facilitation. Biophys. J..

[72] Perez-Reyes E. (2003). Molecular physiology of low-voltage-activated t-type calcium channels. Physiol. Rev..

[73] Crunelli V., Toth T.I., Cope D.W., Blethyn K., Hughes S.W. (2005). The ‘window’ T-type calcium current in brain dynamics of different behavioural states. J. Physiol..

[74] Timic Stamenic T., Todorovic S.M. (2022). Thalamic T-Type Calcium Channels as Targets for Hypnotics and General Anesthetics. Int. J. Mol. Sci..

[75] Talley E.M., Cribbs L.L., Lee J.H., Daud A., Perez-Reyes E., Bayliss D.A. (1999). Differential distribution of three members of a gene family encoding low voltage-activated (T-type) calcium channels. J. Neurosci..

[76] Simms B.A., Zamponi G.W. (2012). Trafficking and stability of voltage-gated calcium channels. Cell Mol. Life Sci..

[77] Wheeler D.G., Groth R.D., Ma H., Barrett C.F., Owen S.F., Safa P., Tsien R.W. (2012). Ca_v_1 and Ca_v_2 channels engage distinct modes of Ca^2+^ signaling to control CREB-dependent gene expression. Cell.

[78] Bannister R.A., Beam K.G. (2013). Impaired gating of an L-Type Ca^2+^ channel carrying a mutation linked to malignant hyperthermia. Biophys. J..

[79] Berridge M.J. (1998). Neuronal calcium signaling. Neuron.

[80] Huguenard J.R. (1996). Low-threshold calcium currents in central nervous system neurons. Annu Rev. Physiol..

[81] Nilius B., Carbone E. (2014). Amazing T-type calcium channels: Updating functional properties in health and disease. Pflugers Arch..

[82] Nilius B., Talavera K., Verkhratsky A. (2006). T-type calcium channels: The never ending story. Cell Calcium..

[83] Bourinet E., Altier C., Hildebrand M.E., Trang T., Salter M.W., Zamponi G.W. (2014). Calcium-permeable ion channels in pain signaling. Physiol. Rev..

[84] Schampel A., Kuerten S. (2017). Danger: High Voltage-The Role of Voltage-Gated Calcium Channels in Central Nervous System Pathology. Cells.

[85] Huguenard J.R., Prince D.A. (1994). Intrathalamic rhythmicity studied in vitro: Nominal T-current modulation causes robust antioscillatory effects. J. Neurosci..

[86] Kim D., Park D., Choi S., Lee S., Sun M., Kim C., Shin H.S. (2003). Thalamic control of visceral nociception mediated by T-type Ca^2+^ channels. Science.

[87] Kim D., Song I., Keum S., Lee T., Jeong M.J., Kim S.S., McEnery M.W., Shin H.S. (2001). Lack of the burst firing of thalamocortical relay neurons and resistance to absence seizures in mice lacking alpha1G T-type Ca^2+^ channels. Neuron.

[88] Anderson M.P., Mochizuki T., Xie J., Fischler W., Manger J.P., Talley E.M., Scammell T.E., Tonegawa S. (2005). Thalamic Ca_v_3.1 T-type Ca^2+^ channel plays a crucial role in stabilizing sleep. Proc. Natl. Acad. Sci. USA.

[89] Bourinet E., Alloui A., Monteil A., Barrere C., Couette B., Poirot O., Pages A., McRory J., Snutch T.P., Eschalier A. (2005). Silencing of the Ca_v_3.2 T-type calcium channel gene in sensory neurons demonstrates its major role in nociception. EMBO J..

[90] Choi S., Na H.S., Kim J., Lee J., Lee S., Kim D., Park J., Chen C.C., Campbell K.P., Shin H.S. (2007). Attenuated pain responses in mice lacking Ca_v_3.2 T-type channels. Genes Brain Behav..

[91] Uebele V.N., Gotter A.L., Nuss C.E., Kraus R.L., Doran S.M., Garson S.L., Reiss D.R., Li Y., Barrow J.C., Reger T.S. (2009). Antagonism of T-type calcium channels inhibits high-fat diet-induced weight gain in mice. J. Clin. Investig..

[92] Uebele V.N., Nuss C.E., Santarelli V.P., Garson S.L., Kraus R.L., Barrow J.C., Stauffer S.R., Koblan K.S., Renger J.J., Aton S. (2009). T-type calcium channels regulate cortical plasticity in-vivo. Neuroreport.

[93] Miwa H., Koh J., Kajimoto Y., Kondo T. (2011). Effects of T-type calcium channel blockers on a parkinsonian tremor model in rats. Pharmacol. Biochem. Behav..

[94] Miwa H., Kondo T. (2011). T-type calcium channel as a new therapeutic target for tremor. Cerebellum.

[95] Francois A., Kerckhove N., Meleine M., Alloui A., Barrere C., Gelot A., Uebele V.N., Renger J.J., Eschalier A., Ardid D. (2013). State-dependent properties of a new T-type calcium channel blocker enhance Ca_v_3.2 selectivity and support analgesic effects. Pain.

[96] Park Y.G., Kim J., Kim D. (2013). The potential roles of T-type Ca^2+^ channels in motor coordination. Front. Neural Circuits.

[97] Yoshimura Y., Inaba M., Yamada K., Kurotani T., Begum T., Reza F., Maruyama T., Komatsu Y. (2008). Involvement of T-type Ca^2+^ channels in the potentiation of synaptic and visual responses during the critical period in rat visual cortex. Eur. J. Neurosci..

[98] Chen C.C., Shen J.W., Chung N.C., Min M.Y., Cheng S.J., Liu I.Y. (2012). Retrieval of context-associated memory is dependent on the Ca_v_3.2 T-type calcium channel. PLoS ONE.

[99] Ly R., Bouvier G., Schonewille M., Arabo A., Rondi-Reig L., Lena C., Casado M., De Zeeuw C.I., Feltz A. (2013). T-type channel blockade impairs long-term potentiation at the parallel fiber-Purkinje cell synapse and cerebellar learning. Proc. Natl. Acad. Sci. USA.

[100] Tang A.H., Karson M.A., Nagode D.A., McIntosh J.M., Uebele V.N., Renger J.J., Klugmann M., Milner T.A., Alger B.E. (2011). Nerve terminal nicotinic acetylcholine receptors initiate quantal GABA release from perisomatic interneurons by activating axonal T-type (Ca_v_3) Ca^2+^ channels and Ca^2+^ release from stores. J. Neurosci..

[101] Weiss N., Hameed S., Fernandez-Fernandez J.M., Fablet K., Karmazinova M., Poillot C., Proft J., Chen L., Bidaud I., Monteil A. (2012). A Ca_v_3.2/syntaxin-1A signaling complex controls T-type channel activity and low-threshold exocytosis. J. Biol. Chem..

[102] Weiss N., Zamponi G.W., De Waard M. (2012). How do T-type calcium channels control low-threshold exocytosis?. Commun. Integr. Biol..

[103] Splawski I., Yoo D.S., Stotz S.C., Cherry A., Clapham D.E., Keating M.T. (2006). CACNA1H mutations in autism spectrum disorders. J. Biol. Chem..

[104] Shcheglovitov A., Zhelay T., Vitko Y., Osipenko V., Perez-Reyes E., Kostyuk P., Shuba Y. (2005). Contrasting the effects of nifedipine on subtypes of endogenous and recombinant T-type Ca^2+^ channels. Biochem. Pharmacol..

[105] Williams M.E., Washburn M.S., Hans M., Urrutia A., Brust P.F., Prodanovich P., Harpold M.M., Stauderman K.A. (1999). Structure and functional characterization of a novel human low-voltage activated calcium channel. J. Neurochem..

[106] Choe W., Messinger R.B., Leach E., Eckle V.S., Obradovic A., Salajegheh R., Jevtovic-Todorovic V., Todorovic S.M. (2011). TTA-P2 is a potent and selective blocker of T-type calcium channels in rat sensory neurons and a novel antinociceptive agent. Mol. Pharmacol..

[107] Sidach S.S., Mintz I.M. (2002). Kurtoxin, a gating modifier of neuronal high- and low-threshold ca channels. J. Neurosci..

[108] Tringham E., Powell K.L., Cain S.M., Kuplast K., Mezeyova J., Weerapura M., Eduljee C., Jiang X., Smith P., Morrison J.L. (2012). T-type calcium channel blockers that attenuate thalamic burst firing and suppress absence seizures. Sci. Transl. Med..

[109] Xiang Z., Thompson A.D., Brogan J.T., Schulte M.L., Melancon B.J., Mi D., Lewis L.M., Zou B., Yang L., Morrison R. (2011). The Discovery and Characterization of ML218: A Novel, Centrally Active T-Type Calcium Channel Inhibitor with Robust Effects in STN Neurons and in a Rodent Model of Parkinson’s Disease. ACS Chem. Neurosci..

[110] Martin R.L., Lee J.H., Cribbs L.L., Perez-Reyes E., Hanck D.A. (2000). Mibefradil block of cloned T-type calcium channels. J. Pharmacol. Exp. Ther..

[111] Chuang R.S., Jaffe H., Cribbs L., Perez-Reyes E., Swartz K.J. (1998). Inhibition of T-type voltage-gated calcium channels by a new scorpion toxin. Nat. Neurosci..

[112] Furukawa T., Miura R., Honda M., Kamiya N., Mori Y., Takeshita S., Isshiki T., Nukada T. (2004). Identification of R(-)-isomer of efonidipine as a selective blocker of T-type Ca^2+^ channels. Br. J. Pharmacol..

[113] Chemin J., Monteil A., Perez-Reyes E., Nargeot J., Lory P. (2001). Direct inhibition of T-type calcium channels by the endogenous cannabinoid anandamide. EMBO J..

[114] Jarvis M.F., Scott V.E., McGaraughty S., Chu K.L., Xu J., Niforatos W., Milicic I., Joshi S., Zhang Q., Xia Z. (2014). A peripherally acting, selective T-type calcium channel blocker, ABT-639, effectively reduces nociceptive and neuropathic pain in rats. Biochem. Pharmacol..

[115] Santi C.M., Cayabyab F.S., Sutton K.G., McRory J.E., Mezeyova J., Hamming K.S., Parker D., Stea A., Snutch T.P. (2002). Differential inhibition of T-type calcium channels by neuroleptics. J. Neurosci..

[116] Atluri N., Joksimovic S.M., Oklopcic A., Milanovic D., Klawitter J., Eggan P., Krishnan K., Covey D.F., Todorovic S.M., Jevtovic-Todorovic V. (2018). A neurosteroid analogue with T-type calcium channel blocking properties is an effective hypnotic, but is not harmful to neonatal rat brain. Br. J. Anaesth.

[117] Monteil A., Chemin J., Leuranguer V., Altier C., Mennessier G., Bourinet E., Lory P., Nargeot J. (2000). Specific properties of T-type calcium channels generated by the human alpha 1I subunit. J. Biol. Chem..

[118] Monteil A., Chemin J., Bourinet E., Mennessier G., Lory P., Nargeot J. (2000). Molecular and functional properties of the human alpha1G subunit that forms T-type calcium channels. J. Biol. Chem..

[119] Kopecky B.J., Liang R., Bao J. (2014). T-type calcium channel blockers as neuroprotective agents. Pflugers Arch..

[120] Li M., Hansen J.B., Huang L., Keyser B.M., Taylor J.T. (2005). Towards selective antagonists of T-type calcium channels: Design, characterization and potential applications of NNC 55-0396. Cardiovasc. Drug Rev..

[121] Moriguchi S., Shioda N., Yamamoto Y., Tagashira H., Fukunaga K. (2012). The T-type voltage-gated calcium channel as a molecular target of the novel cognitive enhancer ST101: Enhancement of long-term potentiation and CaMKII autophosphorylation in rat cortical slices. J. Neurochem..

[122] Yabuki Y., Jing X., Fukunaga K. (2017). The T-type calcium channel enhancer SAK3 inhibits neuronal death following transient brain ischemia via nicotinic acetylcholine receptor stimulation. Neurochem. Int..

[123] Vitko I., Chen Y., Arias J.M., Shen Y., Wu X.R., Perez-Reyes E. (2005). Functional characterization and neuronal modeling of the effects of childhood absence epilepsy variants of CACNA1H, a T-type calcium channel. J. Neurosci..

[124] Murbartian J., Arias J.M., Perez-Reyes E. (2004). Functional impact of alternative splicing of human T-type Ca_v_3.3 calcium channels. J. Neurophysiol..

[125] Coulter D.A., Huguenard J.R., Prince D.A. (1989). Calcium currents in rat thalamocortical relay neurones: Kinetic properties of the transient, low-threshold current. J. Physiol..

[126] Emerick M.C., Stein R., Kunze R., McNulty M.M., Regan M.R., Hanck D.A., Agnew W.S. (2006). Profiling the array of Ca_v_3.1 variants from the human T-type calcium channel gene CACNA1G: Alternative structures, developmental expression, and biophysical variations. Proteins.

[127] Nelson M.T., Joksovic P.M., Perez-Reyes E., Todorovic S.M. (2005). The endogenous redox agent L-cysteine induces T-type Ca^2+^ channel-dependent sensitization of a novel subpopulation of rat peripheral nociceptors. J. Neurosci..

[128] Zhong X., Liu J.R., Kyle J.W., Hanck D.A., Agnew W.S. (2006). A profile of alternative RNA splicing and transcript variation of CACNA1H, a human T-channel gene candidate for idiopathic generalized epilepsies. Hum. Mol. Genet..

[129] Gangadharan G., Shin J., Kim S.W., Kim A., Paydar A., Kim D.S., Miyazaki T., Watanabe M., Yanagawa Y., Kim J. (2016). Medial septal GABAergic projection neurons promote object exploration behavior and type 2 theta rhythm. Proc. Natl. Acad. Sci. USA.

[130] Steriade M., Llinas R.R. (1988). The functional states of the thalamus and the associated neuronal interplay. Physiol. Rev..

[131] Park C., Kim J.H., Yoon B.E., Choi E.J., Lee C.J., Shin H.S. (2010). T-type channels control the opioidergic descending analgesia at the low threshold-spiking GABAergic neurons in the periaqueductal gray. Proc. Natl. Acad. Sci. USA.

[132] Cheong E., Shin H.S. (2013). T-type Ca^2+^ channels in normal and abnormal brain functions. Physiol. Rev..

[133] Cheong E., Shin H.S. (2013). T-type Ca^2+^ channels in absence epilepsy. Biochim. Biophys. Acta.

[134] Freund T.F., Antal M. (1988). GABA-containing neurons in the septum control inhibitory interneurons in the hippocampus. Nature.

[135] Smythe J.W., Colom L.V., Bland B.H. (1992). The extrinsic modulation of hippocampal theta depends on the coactivation of cholinergic and GABA-ergic medial septal inputs. Neurosci. Biobehav. Rev..

[136] Aguado C., Garcia-Madrona S., Gil-Minguez M., Lujan R. (2016). Ontogenic Changes and Differential Localization of T-type Ca^2+^ Channel Subunits Ca_v_3.1 and Ca_v_3.2 in Mouse Hippocampus and Cerebellum. Front. Neuroanat..

[137] Gangarossa G., Laffray S., Bourinet E., Valjent E. (2014). T-type calcium channel Ca_v_3.2 deficient mice show elevated anxiety, impaired memory and reduced sensitivity to psychostimulants. Front. Behav. Neurosci..

[138] Arshaad M.I., Siwek M.E., Henseler C., Daubner J., Ehninger D., Hescheler J., Sachinidis A., Broich K., Papazoglou A., Weiergraber M. (2021). Enhanced hippocampal type II theta activity AND altered theta architecture in mice lacking the Ca_v_3.2 T-type voltage-gated calcium channel. Sci. Rep..

[139] Hara K., Harris R.A. (2002). The anesthetic mechanism of urethane: The effects on neurotransmitter-gated ion channels. Anesth. Analg..

[140] Sceniak M.P., Maciver M.B. (2006). Cellular actions of urethane on rat visual cortical neurons in vitro. J. Neurophysiol..

[141] Papazoglou A., Henseler C., Lundt A., Wormuth C., Soos J., Broich K., Ehninger D., Weiergraber M. (2017). Gender specific hippocampal whole genome transcriptome data from mice lacking the Ca_v_2.3 R-type or Ca_v_3.2 T-type voltage-gated calcium channel. Data Briefs.

[142] Valenzuela J.I., Jaureguiberry-Bravo M., Salas D.A., Ramirez O.A., Cornejo V.H., Lu H.E., Blanpied T.A., Couve A. (2014). Transport along the dendritic endoplasmic reticulum mediates the trafficking of GABAB receptors. J. Cell Sci..

[143] Nakamura T., Arima-Yoshida F., Sakaue F., Nasu-Nishimura Y., Takeda Y., Matsuura K., Akshoomoff N., Mattson S.N., Grossfeld P.D., Manabe T. (2016). PX-RICS-deficient mice mimic autism spectrum disorder in Jacobsen syndrome through impaired GABAA receptor trafficking. Nat. Commun..

[144] Zapata J., Moretto E., Hannan S., Murru L., Longatti A., Mazza D., Benedetti L., Fossati M., Heise C., Ponzoni L. (2017). Epilepsy and intellectual disability linked protein Shrm4 interaction with GABABRs shapes inhibitory neurotransmission. Nat. Commun..

[145] Capogna M., Pearce R.A. (2011). GABAA, slow: Causes and consequences. Trends Neurosci..

[146] Mody I., Pearce R.A. (2004). Diversity of inhibitory neurotransmission through GABA(A) receptors. Trends Neurosci..

[147] Belelli D., Lambert J.J. (2005). Neurosteroids: Endogenous regulators of the GABA(A) receptor. Nat. Rev. Neurosci..

[148] Farrant M., Nusser Z. (2005). Variations on an inhibitory theme: Phasic and tonic activation of GABA(A) receptors. Nat. Rev. Neurosci..

[149] Perez-Garci E., Gassmann M., Bettler B., Larkum M.E. (2006). The GABAB1b isoform mediates long-lasting inhibition of dendritic Ca^2+^ spikes in layer 5 somatosensory pyramidal neurons. Neuron.

[150] Vigot R., Barbieri S., Brauner-Osborne H., Turecek R., Shigemoto R., Zhang Y.P., Lujan R., Jacobson L.H., Biermann B., Fritschy J.M. (2006). Differential compartmentalization and distinct functions of GABAB receptor variants. Neuron.

[151] Bakker R., Tiesinga P., Kotter R. (2015). The Scalable Brain Atlas: Instant Web-Based Access to Public Brain Atlases and Related Content. Neuroinformatics.

[152] Selkoe D.J., Hardy J. (2016). The amyloid hypothesis of Alzheimer’s disease at 25 years. EMBO Mol. Med..

[153] Muller U.C., Deller T., Korte M. (2017). Not just amyloid: Physiological functions of the amyloid precursor protein family. Nat. Rev. Neurosci..

[154] Tang B.L. (2019). Amyloid Precursor Protein (APP) and GABAergic Neurotransmission. Cells.

[155] Sanabria-Castro A., Alvarado-Echeverria I., Monge-Bonilla C. (2017). Molecular Pathogenesis of Alzheimer’s Disease: An Update. Ann. Neurosci..

[156] Fan L., Mao C., Hu X., Zhang S., Yang Z., Hu Z., Sun H., Fan Y., Dong Y., Yang J. (2019). New Insights Into the Pathogenesis of Alzheimer’s Disease. Front. Neurol..

[157] Fan C., Chen K., Zhou J., Wong P.P., He D., Huang Y., Wang X., Ling T., Yang Y., Zhao H. (2021). Systematic analysis to identify transcriptome-wide dysregulation of Alzheimer’s disease in genes and isoforms. Hum. Genet..

[158] Tiwari S., Atluri V., Kaushik A., Yndart A., Nair M. (2019). Alzheimer’s disease: Pathogenesis, diagnostics, and therapeutics. Int. J. Nanomed..

[159] Guo T., Zhang D., Zeng Y., Huang T.Y., Xu H., Zhao Y. (2020). Molecular and cellular mechanisms underlying the pathogenesis of Alzheimer’s disease. Mol. Neurodegener..

[160] Knopman D.S., Amieva H., Petersen R.C., Chetelat G., Holtzman D.M., Hyman B.T., Nixon R.A., Jones D.T. (2021). Alzheimer disease. Nat. Rev. Dis. Primers.

[161] Fanutza T., Del Prete D., Ford M.J., Castillo P.E., D’Adamio L. (2015). APP and APLP2 interact with the synaptic release machinery and facilitate transmitter release at hippocampal synapses. Elife.

[162] Montagna E., Dorostkar M.M., Herms J. (2017). The Role of APP in Structural Spine Plasticity. Front. Mol. Neurosci..

[163] Bruyere J., Abada Y.S., Vitet H., Fontaine G., Deloulme J.C., Ces A., Denarier E., Pernet-Gallay K., Andrieux A., Humbert S. (2020). Presynaptic APP levels and synaptic homeostasis are regulated by Akt phosphorylation of huntingtin. Elife.

[164] Guetg N., Abdel Aziz S., Holbro N., Turecek R., Rose T., Seddik R., Gassmann M., Moes S., Jenoe P., Oertner T.G. (2010). NMDA receptor-dependent GABAB receptor internalization via CaMKII phosphorylation of serine 867 in GABAB1. Proc. Natl. Acad. Sci. USA.

[165] Terunuma M., Pangalos M.N., Moss S.J. (2010). Functional modulation of GABAB receptors by protein kinases and receptor trafficking. Adv. Pharmacol..

[166] Orts-Del’Immagine A., Pugh J.R. (2018). Activity-dependent plasticity of presynaptic GABAB receptors at parallel fiber synapses. Synapse.

[167] Kang J.Y., Chadchankar J., Vien T.N., Mighdoll M.I., Hyde T.M., Mather R.J., Deeb T.Z., Pangalos M.N., Brandon N.J., Dunlop J. (2017). Deficits in the activity of presynaptic gamma-aminobutyric acid type B receptors contribute to altered neuronal excitability in fragile X syndrome. J. Biol. Chem..

[168] Thompson S.E., Ayman G., Woodhall G.L., Jones R.S. (2006). Depression of glutamate and GABA release by presynaptic GABAB receptors in the entorhinal cortex in normal and chronically epileptic rats. Neurosignals.

[169] Borgkvist A., Avegno E.M., Wong M.Y., Kheirbek M.A., Sonders M.S., Hen R., Sulzer D. (2015). Loss of Striatonigral GABAergic Presynaptic Inhibition Enables Motor Sensitization in Parkinsonian Mice. Neuron.

[170] Chu D.C., Penney J.B., Young A.B. (1987). Cortical GABAB and GABAA receptors in Alzheimer’s disease: A quantitative autoradiographic study. Neurology.

[171] Chu D.C., Penney J.B., Young A.B. (1987). Quantitative autoradiography of hippocampal GABAB and GABAA receptor changes in Alzheimer’s disease. Neurosci. Lett..

[172] Iwakiri M., Mizukami K., Ikonomovic M.D., Ishikawa M., Hidaka S., Abrahamson E.E., DeKosky S.T., Asada T. (2005). Changes in hippocampal GABABR1 subunit expression in Alzheimer’s patients: Association with Braak staging. Acta Neuropathol..

[173] Puthiyedth N., Riveros C., Berretta R., Moscato P. (2016). Identification of Differentially Expressed Genes through Integrated Study of Alzheimer’s Disease Affected Brain Regions. PLoS ONE.

[174] Benke D. (2010). Mechanisms of GABAB receptor exocytosis, endocytosis, and degradation. Adv. Pharmacol..

[175] Jo S., Yarishkin O., Hwang Y.J., Chun Y.E., Park M., Woo D.H., Bae J.Y., Kim T., Lee J., Chun H. (2014). GABA from reactive astrocytes impairs memory in mouse models of Alzheimer’s disease. Nat. Med..

[176] Kins S., Lauther N., Szodorai A., Beyreuther K. (2006). Subcellular trafficking of the amyloid precursor protein gene family and its pathogenic role in Alzheimer’s disease. Neurodegener. Dis..

[177] Thinakaran G., Koo E.H. (2008). Amyloid precursor protein trafficking, processing, and function. J. Biol. Chem..

[178] Terunuma M., Vargas K.J., Wilkins M.E., Ramirez O.A., Jaureguiberry-Bravo M., Pangalos M.N., Smart T.G., Moss S.J., Couve A. (2010). Prolonged activation of NMDA receptors promotes dephosphorylation and alters postendocytic sorting of GABAB receptors. Proc. Natl. Acad. Sci. USA.

[179] Amatniek J.C., Hauser W.A., DelCastillo-Castaneda C., Jacobs D.M., Marder K., Bell K., Albert M., Brandt J., Stern Y. (2006). Incidence and predictors of seizures in patients with Alzheimer’s disease. Epilepsia.

[180] Ito K., Tatebe T., Suzuki K., Hirayama T., Hayakawa M., Kubo H., Tomita T., Makino M. (2017). Memantine reduces the production of amyloid-beta peptides through modulation of amyloid precursor protein trafficking. Eur. J. Pharmacol..

[181] Froestl W., Gallagher M., Jenkins H., Madrid A., Melcher T., Teichman S., Mondadori C.G., Pearlman R. (2004). SGS742: The first GABA(B) receptor antagonist in clinical trials. Biochem. Pharmacol..

[182] Gassmann M., Bettler B. (2012). Regulation of neuronal GABA(B) receptor functions by subunit composition. Nat. Rev. Neurosci..

[183] Frangaj A., Fan Q.R. (2018). Structural biology of GABAB receptor. Neuropharmacology.

[184] Hannan S., Gerrow K., Triller A., Smart T.G. (2016). Phospho-dependent Accumulation of GABABRs at Presynaptic Terminals after NMDAR Activation. Cell Rep..

[185] Biermann B., Ivankova-Susankova K., Bradaia A., Abdel Aziz S., Besseyrias V., Kapfhammer J.P., Missler M., Gassmann M., Bettler B. (2010). The Sushi domains of GABAB receptors function as axonal targeting signals. J. Neurosci..

[186] Hannan S., Wilkins M.E., Smart T.G. (2012). Sushi domains confer distinct trafficking profiles on GABAB receptors. Proc. Natl. Acad. Sci. USA.

[187] Dinamarca M.C., Raveh A., Schneider A., Fritzius T., Fruh S., Rem P.D., Stawarski M., Lalanne T., Turecek R., Choo M. (2019). Complex formation of APP with GABAB receptors links axonal trafficking to amyloidogenic processing. Nat. Commun..

[188] Muller U., Kins S. (2002). APP on the move. Trends Mol. Med..

[189] Fu M.M., Holzbaur E.L. (2013). JIP1 regulates the directionality of APP axonal transport by coordinating kinesin and dynein motors. J. Cell Biol..

[190] Eggert S., Thomas C., Kins S., Hermey G. (2018). Trafficking in Alzheimer’s Disease: Modulation of APP Transport and Processing by the Transmembrane Proteins LRP1, SorLA, SorCS1c, Sortilin, and Calsyntenin. Mol. Neurobiol..

[191] Simons M., Ikonen E., Tienari P.J., Cid-Arregui A., Monning U., Beyreuther K., Dotti C.G. (1995). Intracellular routing of human amyloid protein precursor: Axonal delivery followed by transport to the dendrites. J. Neurosci. Res..

[192] Yamazaki T., Selkoe D.J., Koo E.H. (1995). Trafficking of cell surface beta-amyloid precursor protein: Retrograde and transcytotic transport in cultured neurons. J. Cell Biol..

[193] Huang Y., Mucke L. (2012). Alzheimer mechanisms and therapeutic strategies. Cell.

[194] Park M., Salgado J.M., Ostroff L., Helton T.D., Robinson C.G., Harris K.M., Ehlers M.D. (2006). Plasticity-induced growth of dendritic spines by exocytic trafficking from recycling endosomes. Neuron.

[195] Fritzius T., Bettler B. (2020). The organizing principle of GABAB receptor complexes: Physiological and pharmacological implications. Basic. Clin. Pharmacol. Toxicol..

[196] Ring S., Weyer S.W., Kilian S.B., Waldron E., Pietrzik C.U., Filippov M.A., Herms J., Buchholz C., Eckman C.B., Korte M. (2007). The secreted beta-amyloid precursor protein ectodomain APPs alpha is sufficient to rescue the anatomical, behavioral, and electrophysiological abnormalities of APP-deficient mice. J. Neurosci..

[197] Craig M.T., Mayne E.W., Bettler B., Paulsen O., McBain C.J. (2013). Distinct roles of GABAB1a- and GABAB1b-containing GABAB receptors in spontaneous and evoked termination of persistent cortical activity. J. Physiol..

[198] Craig M.T., McBain C.J. (2014). The emerging role of GABAB receptors as regulators of network dynamics: Fast actions from a ‘slow’ receptor?. Curr. Opin. Neurobiol..

[199] Huo Q., Chen M., He Q., Zhang J., Li B., Jin K., Chen X., Long C., Yang L. (2017). Prefrontal Cortical GABAergic Dysfunction Contributes to Aberrant UP-State Duration in APP Knockout Mice. Cereb. Cortex.

[200] Chen C. (2005). beta-Amyloid increases dendritic Ca^2+^ influx by inhibiting the A-type K+ current in hippocampal CA1 pyramidal neurons. Biochem. Biophys. Res. Commun..

[201] Rice R.A., Berchtold N.C., Cotman C.W., Green K.N. (2014). Age-related downregulation of the Ca_v_3.1 T-type calcium channel as a mediator of amyloid beta production. Neurobiol. Aging.

[202] Gavello D., Calorio C., Franchino C., Cesano F., Carabelli V., Carbone E., Marcantoni A. (2018). Early Alterations of Hippocampal Neuronal Firing Induced by Abeta42. Cereb. Cortex.

[203] Kim S., Rhim H. (2011). Effects of amyloid-beta peptides on voltage-gated L-type Ca_v_1.2 and Ca_v_1.3 Ca^2+^ channels. Mol. Cells.

[204] Daschil N., Obermair G.J., Flucher B.E., Stefanova N., Hutter-Paier B., Windisch M., Humpel C., Marksteiner J. (2013). Ca_v_1.2 calcium channel expression in reactive astrocytes is associated with the formation of amyloid-beta plaques in an Alzheimer’s disease mouse model. J. Alzheimers Dis..

[205] Bobich J.A., Zheng Q., Campbell A. (2004). Incubation of nerve endings with a physiological concentration of Abeta1-42 activates Ca_v_2.2 (N-Type)-voltage operated calcium channels and acutely increases glutamate and noradrenaline release. J. Alzheimers Dis..

[206] Hermann D., Mezler M., Muller M.K., Wicke K., Gross G., Draguhn A., Bruehl C., Nimmrich V. (2013). Synthetic Abeta oligomers (Abeta(1-42) globulomer) modulate presynaptic calcium currents: Prevention of Abeta-induced synaptic deficits by calcium channel blockers. Eur. J. Pharmacol..

[207] Sadleir K.R., Popovoic J., Zhu W., Reidel C.T., Do H., Silverman R.B., Vassar R. (2021). Pregabalin Treatment does not Affect Amyloid Pathology in 5XFAD Mice. Curr. Alzheimer Res..

[208] Ishii M., Hiller A.J., Pham L., McGuire M.J., Iadecola C., Wang G. (2019). Amyloid-Beta Modulates Low-Threshold Activated Voltage-Gated L-Type Calcium Channels of Arcuate Neuropeptide Y Neurons Leading to Calcium Dysregulation and Hypothalamic Dysfunction. J. Neurosci..

[209] Zhao Y., Sivaji S., Chiang M.C., Ali H., Zukowski M., Ali S., Kennedy B., Sklyar A., Cheng A., Guo Z. (2017). Amyloid Beta Peptides Block New Synapse Assembly by Nogo Receptor-Mediated Inhibition of T-Type Calcium Channels. Neuron.

[210] Yang L., Wang Z., Wang B., Justice N.J., Zheng H. (2009). Amyloid precursor protein regulates Ca_v_1.2 L-type calcium channel levels and function to influence GABAergic short-term plasticity. J. Neurosci..

[211] Chatzistavraki M., Papazafiri P., Efthimiopoulos S. (2020). Amyloid-beta Protein Precursor Regulates Depolarization-Induced Calcium-Mediated Synaptic Signaling in Brain Slices. J. Alzheimers Dis..

[212] Hefter D., Kaiser M., Weyer S.W., Papageorgiou I.E., Both M., Kann O., Muller U.C., Draguhn A. (2016). Amyloid Precursor Protein Protects Neuronal Network Function after Hypoxia via Control of Voltage-Gated Calcium Channels. J. Neurosci..

[213] Salazar A.M., Leisgang A.M., Ortiz A.A., Murtishaw A.S., Kinney J.W. (2021). Alterations of GABA B receptors in the APP/PS1 mouse model of Alzheimer’s disease. Neurobiol. Aging.

[214] Yang J., Long Y., Xu D.M., Zhu B.L., Deng X.J., Yan Z., Sun F., Chen G.J. (2019). Age- and Nicotine-Associated Gene Expression Changes in the Hippocampus of APP/PS1 Mice. J. Mol. Neurosci..

[215] Resende R., Ferreiro E., Pereira C., Resende de Oliveira C. (2008). Neurotoxic effect of oligomeric and fibrillar species of amyloid-beta peptide 1-42: Involvement of endoplasmic reticulum calcium release in oligomer-induced cell death. Neuroscience.

[216] Demuro A., Smith M., Parker I. (2011). Single-channel Ca^2+^ imaging implicates Abeta1-42 amyloid pores in Alzheimer’s disease pathology. J. Cell Biol..

[217] Green K.N., Demuro A., Akbari Y., Hitt B.D., Smith I.F., Parker I., LaFerla F.M. (2008). SERCA pump activity is physiologically regulated by presenilin and regulates amyloid beta production. J. Gen Physiol..

[218] Oules B., Del Prete D., Greco B., Zhang X., Lauritzen I., Sevalle J., Moreno S., Paterlini-Brechot P., Trebak M., Checler F. (2012). Ryanodine receptor blockade reduces amyloid-beta load and memory impairments in Tg2576 mouse model of Alzheimer disease. J. Neurosci..

[219] Popugaeva E. (2020). Fine Tuning of Intracellular Ca(2+) Content by Pharmacological Agents—A Strategy to Prevent Synapse Loss in Alzheimer Disease Hippocampal Neurons. Curr. Alzheimer Res..

[220] Popugaeva E., Chernyuk D., Bezprozvanny I. (2020). Reversal of Calcium Dysregulation as Potential Approach for Treating Alzheimer’s Disease. Curr. Alzheimer Res..

[221] Anekonda T.S., Quinn J.F. (2011). Calcium channel blocking as a therapeutic strategy for Alzheimer’s disease: The case for isradipine. Biochim. Biophys. Acta.

[222] Anekonda T.S., Quinn J.F., Harris C., Frahler K., Wadsworth T.L., Woltjer R.L. (2011). L-type voltage-gated calcium channel blockade with isradipine as a therapeutic strategy for Alzheimer’s disease. Neurobiol. Dis..

[223] Yagami T., Kohma H., Yamamoto Y. (2012). L-type voltage-dependent calcium channels as therapeutic targets for neurodegenerative diseases. Curr. Med. Chem..

[224] Goodison W.V., Frisardi V., Kehoe P.G. (2012). Calcium channel blockers and Alzheimer’s disease: Potential relevance in treatment strategies of metabolic syndrome. J. Alzheimers Dis..

[225] Cataldi M. (2013). The changing landscape of voltage-gated calcium channels in neurovascular disorders and in neurodegenerative diseases. Curr. Neuropharmacol..

[226] Nimmrich V., Eckert A. (2013). Calcium channel blockers and dementia. Br. J. Pharmacol..

[227] Peters R., Booth A., Peters J. (2014). A systematic review of calcium channel blocker use and cognitive decline/dementia in the elderly. J. Hypertens..

[228] Saravanaraman P., Chinnadurai R.K., Boopathy R. (2014). Why calcium channel blockers could be an elite choice in the treatment of Alzheimer’s disease: A comprehensive review of evidences. Rev. Neurosci..

[229] Lovell M.A., Abner E., Kryscio R., Xu L., Fister S.X., Lynn B.C. (2015). Calcium Channel Blockers, Progression to Dementia, and Effects on Amyloid Beta Peptide Production. Oxid. Med. Cell Longev..

[230] Lopez-Arrieta J.M., Birks J. (2000). Nimodipine for primary degenerative, mixed and vascular dementia. Cochrane Database Syst. Rev..

[231] Wagner G., Icks A., Abholz H.H., Schroder-Bernhardi D., Rathmann W., Kostev K. (2012). Antihypertensive treatment and risk of dementia: A retrospective database study. Int. J. Clin. Pharmacol. Ther..

[232] Liu J., Wang L.N. (2021). Treatment of epilepsy for people with Alzheimer’s disease. Cochrane Database Syst. Rev..

[233] Seibert M., Muhlbauer V., Holbrook J., Voigt-Radloff S., Brefka S., Dallmeier D., Denkinger M., Schonfeldt-Lecuona C., Kloppel S., von Arnim C.A.F. (2021). Efficacy and safety of pharmacotherapy for Alzheimer’s disease and for behavioural and psychological symptoms of dementia in older patients with moderate and severe functional impairments: A systematic review of controlled trials. Alzheimers Res. Ther..

[234] Tariot P.N., Erb R., Leibovici A., Podgorski C.A., Cox C., Asnis J., Kolassa J., Irvine C. (1994). Carbamazepine treatment of agitation in nursing home patients with dementia: A preliminary study. J. Am. Geriatr. Soc..

[235] Tariot P.N., Erb R., Podgorski C.A., Cox C., Patel S., Jakimovich L., Irvine C. (1998). Efficacy and tolerability of carbamazepine for agitation and aggression in dementia. Am. J. Psychiatry.

[236] Olin J.T., Fox L.S., Pawluczyk S., Taggart N.A., Schneider L.S. (2001). A pilot randomized trial of carbamazepine for behavioral symptoms in treatment-resistant outpatients with Alzheimer disease. Am. J. Geriatr. Psychiatry.

[237] Porsteinsson A.P., Tariot P.N., Erb R., Cox C., Smith E., Jakimovich L., Noviasky J., Kowalski N., Holt C.J., Irvine C. (2001). Placebo-controlled study of divalproex sodium for agitation in dementia. Am. J. Geriatr. Psychiatry.

[238] Dogrul A., Gardell L.R., Ossipov M.H., Tulunay F.C., Lai J., Porreca F. (2003). Reversal of experimental neuropathic pain by T-type calcium channel blockers. Pain.

[239] Oshima T., Ozono R., Yano Y., Higashi Y., Teragawa H., Miho N., Ishida T., Ishida M., Yoshizumi M., Kambe M. (2005). Beneficial effect of T-type calcium channel blockers on endothelial function in patients with essential hypertension. Hypertens. Res..

[240] Astori S., Wimmer R.D., Prosser H.M., Corti C., Corsi M., Liaudet N., Volterra A., Franken P., Adelman J.P., Luthi A. (2011). The Ca_v_3.3 calcium channel is the major sleep spindle pacemaker in thalamus. Proc. Natl. Acad. Sci. USA.

[241] Zamponi G.W. (2016). Targeting voltage-gated calcium channels in neurological and psychiatric diseases. Nat. Rev. Drug Discov..

[242] Cai S., Gomez K., Moutal A., Khanna R. (2021). Targeting T-type/Ca_v_3.2 channels for chronic pain. Transl. Res..

[243] Lei D., Gao X., Perez P., Ohlemiller K.K., Chen C.C., Campbell K.P., Hood A.Y., Bao J. (2011). Anti-epileptic drugs delay age-related loss of spiral ganglion neurons via T-type calcium channel. Hear. Res..

[244] Beghi E., Beghi M. (2020). Epilepsy, antiepileptic drugs and dementia. Curr. Opin. Neurol..

[245] Taipale H., Gomm W., Broich K., Maier W., Tolppanen A.M., Tanskanen A., Tiihonen J., Hartikainen S., Haenisch B. (2018). Use of Antiepileptic Drugs and Dementia Risk-an Analysis of Finnish Health Register and German Health Insurance Data. J. Am. Geriatr. Soc..

[246] Toyota M., Ho C., Ohe-Toyota M., Baylin S.B., Issa J.P. (1999). Inactivation of CACNA1G, a T-type calcium channel gene, by aberrant methylation of its 5’ CpG island in human tumors. Cancer Res..

[247] Garcia-Baquero R., Puerta P., Beltran M., Alvarez M., Sacristan R., Alvarez-Ossorio J.L., Sanchez-Carbayo M. (2013). Methylation of a novel panel of tumor suppressor genes in urine moves forward noninvasive diagnosis and prognosis of bladder cancer: A 2-center prospective study. J. Urol..

[248] Green K.N., Khashwji H., Estrada T., Laferla F.M. (2011). ST101 induces a novel 17 kDa APP cleavage that precludes Abeta generation in vivo. Ann. Neurol..

[249] Gauthier S., Rountree S., Finn B., LaPlante B., Weber E., Oltersdorf T. (2015). Effects of the Acetylcholine Release Agent ST101 with Donepezil in Alzheimer’s Disease: A Randomized Phase 2 Study. J. Alzheimers Dis..

[250] Fukunaga K., Izumi H., Yabuki Y., Shinoda Y., Shioda N., Han F. (2019). Alzheimer’s disease therapeutic candidate SAK3 is an enhancer of T-type calcium channels. J. Pharmacol. Sci..

[251] Izumi H., Shinoda Y., Saito T., Saido T.C., Sato K., Yabuki Y., Matsumoto Y., Kanemitsu Y., Tomioka Y., Abolhassani N. (2018). The Disease-modifying Drug Candidate, SAK3 Improves Cognitive Impairment and Inhibits Amyloid beta Deposition in App Knock-in Mice. Neuroscience.

[252] Cilz N.I., Kurada L., Hu B., Lei S. (2014). Dopaminergic modulation of GABAergic transmission in the entorhinal cortex: Concerted roles of alpha1 adrenoreceptors, inward rectifier K^+^, and T-type Ca^2+^ channels. Cereb. Cortex.

[253] Liu X.B., Murray K.D., Jones E.G. (2011). Low-threshold calcium channel subunit Ca_v_3.3 is specifically localized in GABAergic neurons of rodent thalamus and cerebral cortex. J. Comp. Neurol..

[254] Takeda K., Yamaguchi Y., Hino M., Kato F. (2016). Potentiation of Acetylcholine-Mediated Facilitation of Inhibitory Synaptic Transmission by an Azaindolizione Derivative, ZSET1446 (ST101), in the Rat Hippocampus. J. Pharmacol. Exp. Ther..

[255] Park H.J., Kim S.S., Kang S., Rhim H. (2009). Intracellular Abeta and C99 aggregates induce mitochondria-dependent cell death in human neuroglioma H4 cells through recruitment of the 20S proteasome subunits. Brain Res..

[256] Hoshi M., Sato M., Matsumoto S., Noguchi A., Yasutake K., Yoshida N., Sato K. (2003). Spherical aggregates of beta-amyloid (amylospheroid) show high neurotoxicity and activate tau protein kinase I/glycogen synthase kinase-3beta. Proc. Natl. Acad. Sci. USA.

[257] Gregori L., Fuchs C., Figueiredo-Pereira M.E., Van Nostrand W.E., Goldgaber D. (1995). Amyloid beta-protein inhibits ubiquitin-dependent protein degradation in vitro. J. Biol. Chem..

[258] Keck S., Nitsch R., Grune T., Ullrich O. (2003). Proteasome inhibition by paired helical filament-tau in brains of patients with Alzheimer’s disease. J. Neurochem..

[259] Dantuma N.P., Bott L.C. (2014). The ubiquitin-proteasome system in neurodegenerative diseases: Precipitating factor, yet part of the solution. Front. Mol. Neurosci..

[260] Ross J.M., Olson L., Coppotelli G. (2015). Mitochondrial and Ubiquitin Proteasome System Dysfunction in Ageing and Disease: Two Sides of the Same Coin?. Int. J. Mol. Sci..

[261] Gadhave K., Bolshette N., Ahire A., Pardeshi R., Thakur K., Trandafir C., Istrate A., Ahmed S., Lahkar M., Muresanu D.F. (2016). The ubiquitin proteasomal system: A potential target for the management of Alzheimer’s disease. J. Cell Mol. Med..

[262] Huang L., Keyser B.M., Tagmose T.M., Hansen J.B., Taylor J.T., Zhuang H., Zhang M., Ragsdale D.S., Li M. (2004). NNC 55-0396 [(1S,2S)-2-(2-(N-[(3-benzimidazol-2-yl)propyl]-N-methylamino)ethyl)-6-fluoro-1,2, 3,4-tetrahydro-1-isopropyl-2-naphtyl cyclopropanecarboxylate dihydrochloride]: A new selective inhibitor of T-type calcium channels. J. Pharmacol. Exp. Ther..

[263] Santarelli L., Saxe M., Gross C., Surget A., Battaglia F., Dulawa S., Weisstaub N., Lee J., Duman R., Arancio O. (2003). Requirement of hippocampal neurogenesis for the behavioral effects of antidepressants. Science.

[264] Shioda N., Yamamoto Y., Han F., Moriguchi S., Yamaguchi Y., Hino M., Fukunaga K. (2010). A novel cognitive enhancer, ZSET1446/ST101, promotes hippocampal neurogenesis and ameliorates depressive behavior in olfactory bulbectomized mice. J. Pharmacol. Exp. Ther..

[265] Bao H., Asrican B., Li W., Gu B., Wen Z., Lim S.A., Haniff I., Ramakrishnan C., Deisseroth K., Philpot B. (2017). Long-Range GABAergic Inputs Regulate Neural Stem Cell Quiescence and Control Adult Hippocampal Neurogenesis. Cell Stem Cell.

[266] Campbell N.R., Fernandes C.C., Halff A.W., Berg D.K. (2010). Endogenous signaling through alpha7-containing nicotinic receptors promotes maturation and integration of adult-born neurons in the hippocampus. J. Neurosci..

[267] Xu J., Yabuki Y., Yu M., Fukunaga K. (2018). T-type calcium channel enhancer SAK3 produces anti-depressant-like effects by promoting adult hippocampal neurogenesis in olfactory bulbectomized mice. J. Pharmacol. Sci..

[268] Nam G. (2018). T-type calcium channel blockers: A patent review (2012–2018). Expert Opin. Ther. Pat..

[269] Weiss N., Zamponi G.W. (2019). T-type calcium channels: From molecule to therapeutic opportunities. Int. J. Biochem. Cell Biol..

[270] Weiss N., Zamponi G.W. (2019). T-Type Channel Druggability at a Crossroads. ACS Chem. Neurosci..

[271] Giordanetto F., Knerr L., Wallberg A. (2011). T-type calcium channels inhibitors: A patent review. Expert Opin. Ther. Pat..

[272] Gomora J.C., Daud A.N., Weiergraber M., Perez-Reyes E. (2001). Block of cloned human T-type calcium channels by succinimide antiepileptic drugs. Mol. Pharmacol..

[273] Matar N., Jin W., Wrubel H., Hescheler J., Schneider T., Weiergraber M. (2009). Zonisamide block of cloned human T-type voltage-gated calcium channels. Epilepsy Res..

[274] Remy S., Beck H. (2006). Molecular and cellular mechanisms of pharmacoresistance in epilepsy. Brain.

[275] Lacinova L., Klugbauer N., Hofmann F. (2000). Low voltage activated calcium channels: From genes to function. Gen. Physiol. Biophys..

[276] Lacinova L., Klugbauer N., Hofmann F. (2000). State- and isoform-dependent interaction of isradipine with the alpha1C L-type calcium channel. Pflugers Arch..

[277] Lacinova L., Klugbauer N., Hofmann F. (2000). Regulation of the calcium channel alpha1G subunit by divalent cations and organic blockers. Neuropharmacology.

[278] Perchenet L., Clement-Chomienne O. (2000). Characterization of mibefradil block of the human heart delayed rectifier hK_v_1.5. J. Pharmacol. Exp. Ther..

[279] Perchenet L., Benardeau A., Ertel E.A. (2000). Pharmacological properties of Ca_v_3.2, a low voltage-activated Ca^2+^ channel cloned from human heart. Naunyn Schmiedebergs Arch. Pharmacol..

[280] Kumar P.P., Stotz S.C., Paramashivappa R., Beedle A.M., Zamponi G.W., Rao A.S. (2002). Synthesis and evaluation of a new class of nifedipine analogs with T-type calcium channel blocking activity. Mol. Pharmacol..

[281] Flanagan R.J. (1998). Guidelines for the interpretation of analytical toxicology results and unit of measurement conversion factors. Ann. Clin. Biochem..

[282] Freeze B.S., McNulty M.M., Hanck D.A. (2006). State-dependent verapamil block of the cloned human Ca_v_3.1 T-type Ca^2+^ channel. Mol. Pharmacol..

[283] Hainsworth A.H., McNaughton N.C., Pereverzev A., Schneider T., Randall A.D. (2003). Actions of sipatrigine, 202W92 and lamotrigine on R-type and T-type Ca^2+^ channel currents. Eur. J. Pharmacol..

[284] McNaughton N.C., Hainsworth A.H., Green P.J., Randall A.D. (2000). Inhibition of recombinant low-voltage-activated Ca^2+^ channels by the neuroprotective agent BW619C89 (Sipatrigine). Neuropharmacology.

[285] Todorovic S.M., Perez-Reyes E., Lingle C.J. (2000). Anticonvulsants but not general anesthetics have differential blocking effects on different T-type current variants. Mol. Pharmacol..

[286] Lau C., Ng L., Thompson C., Pathak S., Kuan L., Jones A., Hawrylycz M. (2008). Exploration and visualization of gene expression with neuroanatomy in the adult mouse brain. BMC Bioinform..

